# Malaria paediatric hospitalization between 1999 and 2008 across Kenya

**DOI:** 10.1186/1741-7015-7-75

**Published:** 2009-12-09

**Authors:** Emelda A Okiro, Victor A Alegana, Abdisalan M Noor, Juliette J Mutheu, Elizabeth Juma, Robert W Snow

**Affiliations:** 1 Malaria Public Health & Epidemiology Group, Centre for Geographic Medicine Research - Coast, Kenya Medical Research Institute/Wellcome Trust Research Programme, P.O. Box 43640, 00100 GPO, Nairobi, Kenya; 2 Centre for Tropical Medicine, Nuffield Department of Clinical Medicine, University of Oxford, CCVTM, Oxford OX3 7LJ, UK; 3 Division of Malaria Control, Ministry of Public Health and Sanitation, P.O Box 19982, 00202 KNH, Nairobi, Kenya

## Abstract

**Background:**

Intervention coverage and funding for the control of malaria in Africa has increased in recent years, however, there are few descriptions of changing disease burden and the few reports available are from isolated, single site observations or are of reports at country-level. Here we present a nationwide assessment of changes over 10 years in paediatric malaria hospitalization across Kenya.

**Methods:**

Paediatric admission data on malaria and non-malaria diagnoses were assembled for the period 1999 to 2008 from in-patient registers at 17 district hospitals in Kenya and represented the diverse malaria ecology of the country. These data were then analysed using autoregressive moving average time series models with malaria and all-cause admissions as the main outcomes adjusted for rainfall, changes in service use and populations-at-risk within each hospital's catchment to establish whether there has been a statistically significant decline in paediatric malaria hospitalization during the observation period.

**Results:**

Among the 17 hospital sites, adjusted paediatric malaria admissions had significantly declined at 10 hospitals over 10 years since 1999; had significantly increased at four hospitals, and remained unchanged in three hospitals. The overall estimated average reduction in malaria admission rates was 0.0063 cases per 1,000 children aged 0 to 14 years per month representing an average percentage reduction of 49% across the 10 hospitals registering a significant decline by the end of 2008. Paediatric admissions for all-causes had declined significantly with a reduction in admission rates of greater than 0.0050 cases per 1,000 children aged 0 to 14 years per month at 6 of 17 hospitals. Where malaria admissions had increased three of the four sites were located in Western Kenya close to Lake Victoria. Conversely there was an indication that areas with the largest declines in malaria admission rates were areas located along the Kenyan coast and some sites in the highlands of Kenya.

**Conclusion:**

A country-wide assessment of trends in malaria hospitalizations indicates that all is not equal, important variations exist in the temporal pattern of malaria admissions between sites and these differences require more detailed investigation to understand what is required to promote a clinical transition across Africa.

## Background

In recent years several African countries have managed to rapidly scale up the delivery of key malaria control measures, notably insecticide treated nets (ITN) [1] and changing policies to support the provision of efficacious Artemisinin-based combination therapies (ACT) for malaria case-management [2]. Documenting expanding coverage is important to reconcile against international donor and government financial investment, however increasing the numbers of individuals protected and adequately treated must be linked to changes in malaria disease burdens, if future malaria control investment is to be sustained. At several sites across Africa there have been recent attempts to attribute changing patterns of malaria morbidity to scaled intervention coverage [3-14], malaria hospitalization [3-6,9,11,13-18] or malaria-attributable mortality [3-5,7,11,13,14,16,19-21].

The popular perception is that the epidemiology of clinical malaria is in transition across Africa [22-25]. A comprehensive assessment is hampered by the selective reporting of only *good news *stories or the occasional editorial biases toward positive effects in peer-reviewed journals [22]. To date reports of changing disease risks have been limited to observations from selective sites limiting generalisability [5,11,16-18]; or reported at country-levels ignoring the heterogeneity within countries [2,9,14,26]; or inadequately adjusted for missing data, health service use and populations-at-risk [7,9,12,15].

Here we present a country-wide assessment of changing paediatric admissions to 17 district general hospitals across Kenya using novel approaches to adjust for the populations served by these hospitals, general service use and the periodicity effects of rainfall over a 10 year period between 1999 and 2008.

## Methods

### Description of study sites and cluster allocation

Kenya has a diverse malaria ecology ranging from the virtual absence of locally acquired infection to stable, high intensity endemic transmission [27-30]. There are an estimated 181 government supported district and sub-district general hospitals providing in-patient paediatric care services of which 61 are located in areas of almost no malaria transmission in Central, Nairobi and Rift Valley provinces. Among the 120 remaining hospitals, almost none have complete records reported to national Ministry of Health headquarters that can be used for time-series analysis [31]. Incomplete admission records are also a common feature at the hospital level ([31]; unpublished observations). Many hospitals are small facilities, having been up-graded from health centres, and thus not all have dedicated paediatric wards or staff. Against this background, 23 high patient admission hospitals with paediatric wards were identified in consultation with the Ministry of Health to reflect the diversity of malaria transmission across Kenya and likelihood of being able to provide records of admissions over the last 10 years. Six hospitals were subsequently dropped following site visits as they were unable to provide more than 100 of 120 observation months of admission data.

**Figure 1:**
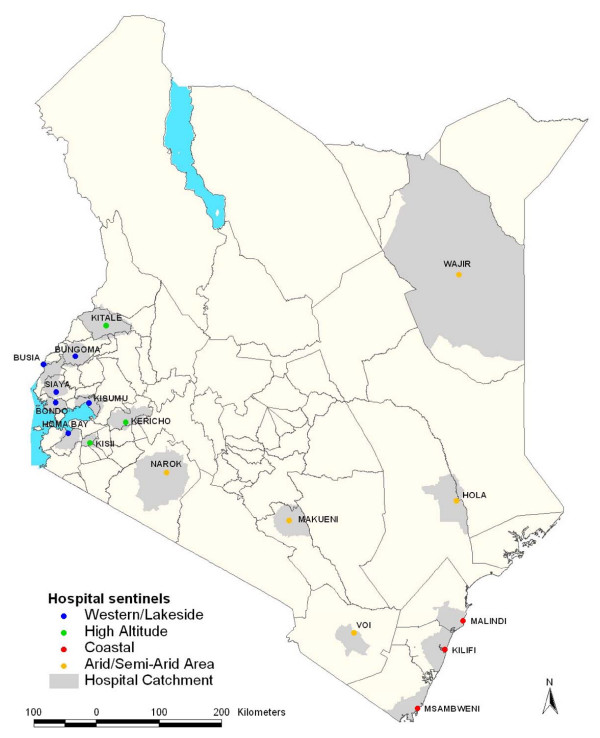
**Map showing the Location of District hospital sites in Kenya colour coded according to region; Coast (red dots), Western/Lakeside (blue dots), Highlands (green dots) and Semi-Arid areas (yellow dots) and the catchment area for each hospital (light grey areas around each hospital)**.

The selected hospital sites are shown in Figure 1 and their malaria ecological characteristics are shown in Table 1. Broadly the hospital sites include six hospitals located in the Western part of Kenya bordering Lake Victoria or the Ugandan border (Busia, Bungoma, Bondo, Homa Bay, Kisumu and Siaya), all areas historically supporting high, intense perennial malaria transmission; three hospitals where the populations live predominantly above 1,500 meters (m) above sea level (Kericho, Kisii, and Kitale); along the Kenyan coast three hospitals (at Kilifi, Malindi and Msambweni) previously described up to 2007 [15]; and five hospitals located in areas that are predominantly arid and have historically low risks of malaria infection (Narok, Hola, Voi, Makueni and Wajir) and where part of the populations at Narok, Voi and Makueni also live above 1,500 m.

**Table 1:** Characteristics of the defined hospital catchment areas communities for 17 hospitals

Hospital location	Total Population 1999 in catchment **[Surface area Km**^2 ^**]**	Projected population <15 years in 2004 (Mid-point of surveillance) [inter-censal growth rate] % p.a.	**% of Population residing in Areas ≥ 1500 m above sea level**^2 ^	% of Population residing in Areas with **EVI <0.3**^3 ^	**Average annual rainfall 1999-2008 (mm)**^4 ^	**% population living in areas with modelled *Pf *PR**_2-10 _**<5% in 2009**	**% Population living in areas with modelled *Pf *PR**_2-10 _**5-39% in 2009**	% Population living in areas with modelled ***Pf *PR**_2-10 _**≥ 40% in 2009**
**Western/Lakeside**								
**Busia**	369,552 [9.3]	204,650 [3.4]	0.0	0.0	1,584	0.0	0.0	100.0
**Bungoma**	569,953 [12.5]	339,862 [4.3]	24.4	0.0	1,431	0.0	53.5	46.5
**Bondo**	153,864 [6.0]	75,862 [1.8]	0.0	1.27	1,412^5 ^	0.0	44.7	55.2
**Homa Bay**	253,496 [8.5]	130,559 [2.7]	0.9	11.0	1,412^5 ^	0.0	77.5	22.5
**Kisumu**	541,002 [8.4]	251,398 [2.0]	13.0	15.8	1,412^5 ^	0.0	89.6	10.4
**Siaya**	243,149 [7.0]	112,039 [0.9]	0.0	0.7	1,412^5 ^	0.0	1.3	98.7
**Highlands**								
**Kericho**	406,491 [15.6]	198,951 [2.4]	55.2	0.00	2,121	84.9	15.3	0.0
**Kisii**	405,331 [4.7]	197,580 [2.0]	88.5	0.00	2,119	10.3	89.7	0.0
**Kitale**	765,943 [26.9]	435,428 [3.8]	84.1	2.8	1,264	69.8	30.2	0.0
**Coastal**								
**Kilifi**	338,112 [17.6]	183,651 [3.1]	0.0	5.3	1,349^6 ^	28.8	71.2	0.0
**Malindi**	201,049 [12.7]	114,865 [3.4]	0.0	18.8	1,077	61.7	38.3	0.0
**Msambweni**	241,647 [14.5]	101,802 [2.6]	0.0	1.15	887 (5)	0.0	93.3	6.8
**Arid & semi-arid**								
**Narok**	167,519 [35.0]	96,886 [3.3]	44.7	33.9	722^7 ^	100.0	0.0	0.0
**Hola**	52,622 [31.3]	29,264 [3.4]	0.0	73.2	492	6.0	94.0	0.0
**Voi**	159,230 [14.3]	69,613 [1.7]	21.9	34.4	491^7 ^	94.8	5.2	0.0
**Makueni**	293,997 [20.4]	159,381 [2.8]	15.2	48.3	540	100.0	0.0	0.0
**Wajir**	287,617 [446.8]^1 ^	224,625 [9.6]	0.0	100.0	312	100.0	0.0	0.0

1. Wajir is a district that is populated by pastoralists with very little access to health services and scattered across a wide spatial area. The predicted catchment area therefore was considerably larger than all other more densely populated settled communities in other districts.

2. A digital elevation map (DEM) for Kenya was used that has a resolution of 30 meters http://www.vterrain.org/Elevation/SRTM/

3. EVI - Enhanced vegetation index - threshold values were computed to define malaria-relevant EVI categories that corresponded to accepted definitions of aridity based on annual rainfall [53]. In Kenya this approximated to an EVI threshold of 0.3 below which average annual rainfall was less than 1000 mm corresponding to historical descriptions of malaria in Kenya referred to as 'only malarious near water' [27].

4. Trend analysis of rainfall showed a relatively stable pattern across most of the sites without significant rises or declines in monthly precipitation over the 10 years of observation. Exceptions were at Msambweni where a significant, but small downward trend was observed (trend 0.44; *P *value for t statistic = 0.018,) and at Hola and Busia significant increased precipitation was observed over the 10 years; trend = 0.27 (*P *= 0.042) and trend = 0.79 (*P *= 0.002) respectively.

5. A single meteorological station was used for all the four hospitals immediately surrounding Lake Victoria, based at Kisumu, this met station had complete records for the period of observation and there were no other met stations located close to the catchments of the other three hospital sites. This station is however located close, between 39 and 54 km, to the other three catchment areas.

6. Rainfall data from the Kilifi Met station were missing for the period June 2008 to December 2008 and were supplemented for this period with data from Malindi met station located 24 km from the Kilifi catchment area.

7. A total of 13 (0.6%) of 2,040 monthly rainfall observations were not recorded. An average of neighbouring non-missing values was used to estimate single months of missing rainfall data in two sites (Voi and Narok).

### Paediatric admission data

Paediatric ward in-patient registers at all hospitals, except Kilifi, were identified for most months from January 1999 to December 2008. Each admission entry in the registers was recorded on a tally sheet indicating the month of admission and whether a primary working diagnosis of malaria had been defined for the child or whether the admission diagnosis did not include an indication of malaria. Exact dates of birth were not available for all admissions and we have assumed all paediatric admissions were aged between 0 and <15 years. Individual register entries were not reconciled with patient notes and thus an assumption was made that the admission diagnosis remained the diagnosis upon which each admission was managed clinically. Slide data confirming malaria diagnoses at admission were not assembled because the completeness of these laboratory procedures is extremely poor in most Kenyan district hospitals and results, when available, are rarely used to refine an admission diagnosis [32]. Hereafter we refer to uncomplicated, malaria admission diagnoses as malaria, accepting that in hospitals without complete parasitology, this diagnosis is only suspected malaria. Kilifi is an exception where parasitologically-confirmed diagnosis was used as the final diagnosis and a comprehensive paediatric ward surveillance system has been operational since 1989 [32-34]. At Kilifi, demographic details and clinical histories are recorded on every admission and a finger prick blood sample is taken for malaria parasitology. Clinical and laboratory findings, clinical progress and response to therapy were reviewed at discharge to derive a primary diagnosis which was recorded on a standard admission proforma and later entered into a centralized database. Data for the present study were reassembled for the period January 1999 to December 2008 and summarized by month as a discharge diagnosis of malaria or non-malaria for all admissions aged between 0 and 15 years of age.

Of a total of 2,040 possible monthly data records for malaria, 45 (2.2%) were not retrieved from the hospital records or were incomplete for a continuous month because pages were torn, unreadable or a whole register was absent. These included seven months from Busia, five months from Bungoma, nine months from Homa Bay, five months from Kisumu and 19 months from Kericho. Missing data were handled in STATA (version 10.1) creating multiple imputed data sets for all missing values using other existing variables in this case rainfall and the number of malaria and non-malaria deaths for each missing time point. An average value was taken to yield a monthly estimate for each missing data point.

### Defining the Health Facility Catchment Population

Catchment areas for each hospital were defined to obtain an estimate of the populations served by the facility in order to standardize rates of admission between hospitals. A patient's residence is routinely entered on hospital in-patient registers and these were reviewed for the months of March, June and October in a single year, allowing for possible seasonal differences in hospital access. The village and/or area name entered into the paediatric registers was transcribed for all admissions during these three months irrespective of admission diagnosis. Village residence was then geo-located using a combination of national databases of villages, schools and other settlement features [35]. Where villages could not be positioned from these sources, centroids of enumeration area (EA) polygons, the smallest population unit used in the 1999 Kenya national census and often an equivalent of a village was used. Data points were mapped in ARCGIS 9.1 (ESRI, Inc., Redland, CA, USA). Initially, the distance at which the utilization began to decline was used as a threshold and all EA populations within a buffer of radius equal to this distance defined. Subsequently, we created the smallest polygon bounding all of the members of an EA set around the outer extent of the furthest villages of in-patients admitted to the hospital using ARCGIS 9.2 and an algorithm was used to select a digitized set of EAs that represented 90% of the spatially positioned admissions. These boundaries of irregular EA polygons were then combined and smoothed using a visual inspection of the defined catchment and imputing missing EAs at the outer limits based on road networks and physical barriers such as rivers, dams and hills [36]. This refined area was designated as the final hospital's catchment area and used for all subsequent analysis and supplementary data assemblies (Additional file 1).

The EA configurations of the catchment area were extracted and matched to the national census counts of total population documented in August 1999 [37]. The total populations in 1999 were then corrected for the average age distributions of sub-locations that covered the catchment areas (between 33 and 111 depending on the district) to estimate the population aged less than 15 years. This medium-variant under 15 years census count was then projected for each of the subsequent ten years of observation using the annualized district growth rates defined in 1999 estimated from inter-censal growth since 1989 [37]. The projected annual hospital catchment populations aged 0 to 14 years were then used to compute rates per 1,000 population-at-risk using monthly malaria and non-malaria admission data in each of the years of review.

### Climate data

Rainfall is one of the most important weather variables that drive inter- and intra-annual periodicity in malaria admissions [38-40]. To adjust for the short and long-term effects of rainfall, monthly precipitation data were obtained from meteorological stations located within the catchment area of 11 hospitals and because of incomplete or unavailable records rainfall data was obtained from the nearest possible metrological station with complete data for six hospital catchment areas (Makueni, Msambweni, Hola, Bondo, Homa Bay, Siaya) ranging from 39 km to 85 km from the catchment boundary.

### Defining parasite prevalence within the catchment area

We have recently reported a spatial-temporal Bayesian generalized linear geo-statistical model that allows for the spatial prediction (1 × 1 km^2 ^) of the *Plasmodium falciparum *parasite prevalence among children aged 2 to 10 years of age (*Pf *PR_2-10 _) in Kenya [30]. These methods assume a basic principal that if survey points are closer in time they are likely to be more similar than if they are further apart in time and if they are closer in space they will also be more similar than if they were many kilometers apart. The models used allow for a prediction in areas where no information exists using the data from neighboring time and space located communities where data do exist and adjusted for the predictive effects of where an un-sampled location is in relation to various relevant covariates. The model was developed using empirical data on parasite prevalence from over 2,500 surveys and uses spatial covariates including urbanization, aridity, distance to permanent water and ambient maximum temperature. We have used the projected predictions here to provide a more informed context of infection risks in each of the predefined catchment areas at the end of the time-series malaria admission period (2009). Within each hospital catchment the percentage of the population exposed to low transmission intensity (*Pf *PR_2-10 _< 5%), moderate transmission intensity (*Pf *PR_2-10 _5-39%) and high intensity transmission (*Pf *PR_2-10 _≥ 40%) were computed.

### Data Analysis

After adjusting all the malaria and non-malaria data for missing values, average annual admission rates were computed for three periods that correspond to important milestones for malaria intervention and drug policy change in Kenya: 1999 to 2002 where ITN coverage was nationally low [41,42] and failing monotherapies were widely available and commonly used; 2003 to 2005 a period when ITN coverage began to marginally increase [42] but resistance to sulphadoxine-pyrimethamine (SP) had escalated to alarming levels [43]; and 2006 to 2008 when national campaigns to deliver ITN free-of-charge massively increased coverage [42] and SP was replaced as the recommended first-line therapy for uncomplicated malaria with Artemether-Lumefathrine but only available in the public health sector [43] characterized by poor and incomplete supply [44] and with poor coverage for febrile children [45]. Confidence intervals around each average annualized admission rate per 1,000 children aged 0 to 14 years were computed using the Poisson distributions [46] to compute exact Poisson confidence intervals.

To examine the long-term trends in admission rates we first used moving average smoothing methods to highlight longer-term trend signals within each temporal 10-year series to filter out short-term annual fluctuations and random variation. The smoothing technique achieves this by replacing each element of the time series by *n *neighbouring elements, where *n *is the width of the smoothing *window *and is equal to 12 months. We employed a centered moving average including six observations before and five after the time point and vice versa and taking an average value of this to compute the centred average. The ARMAX model was then applied [47]. This model is an autoregressive (AR) model of the empirical current value of the series against one or more prior values combined with a moving average (MA) of the current value modeled against the white noise (random shocks) of one or more prior values and includes explanatory variables. The explanatory variables included rainfall in the preceding months and changes in service use (captured by non-malaria admission rates) resulting in a predicted or smoothed malaria admission rate per month for each hospital site over the period 1999 to 2008. Before specifying the ARMAX model, tests and diagnostics were performed in the estimation of the models. Monthly malaria case data were tested for stationarity using the augmented Dickey Fuller test [48] with a lag of 12 months. Insignificant AR and MA lags were excluded and model diagnostics and selection criteria were used to determine the most parsimonious model. Two goodness-of-fit criteria were used to guide model selection: Akaike Information Criterion (AIC) and Schwartz's Bayesian information criterion (BIC). The models with the lowest AIC and BIC were finally selected. All analysis was undertaken using STATA version 10.1 (Statacorp 2003, College Station, Texas, USA).

## Results

**Figure 2:**
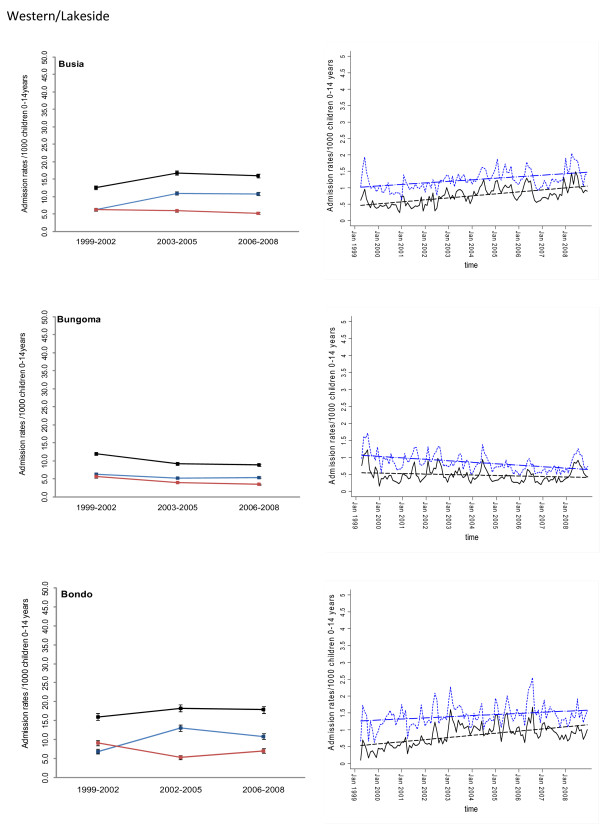
**Plots of paediatric admission data for malaria (blue line) and non-malaria (red line) and all-cause admissions (black line) at three hospitals in Western/Lakeside Region (Busia, Bungoma and Bondo) expressed per 1,000 children aged 0 to 14 years at risk per annum and 95% confidence intervals presented as aggregated data in three time periods: 1999 to 2002, 2003 to 2005 and 2006 to 2008 (Left panels). **Model predictions of all-cause rates controlling for lagged rainfall (dotted blue line) and malaria hospitalization rates controlling for lagged rainfall and non-malaria cases and controlling for autoregressive and moving average effects (solid black line). Fitted lines illustrate the linear trends from model predictions (dashed line) (right panel).

**Figure 3:**
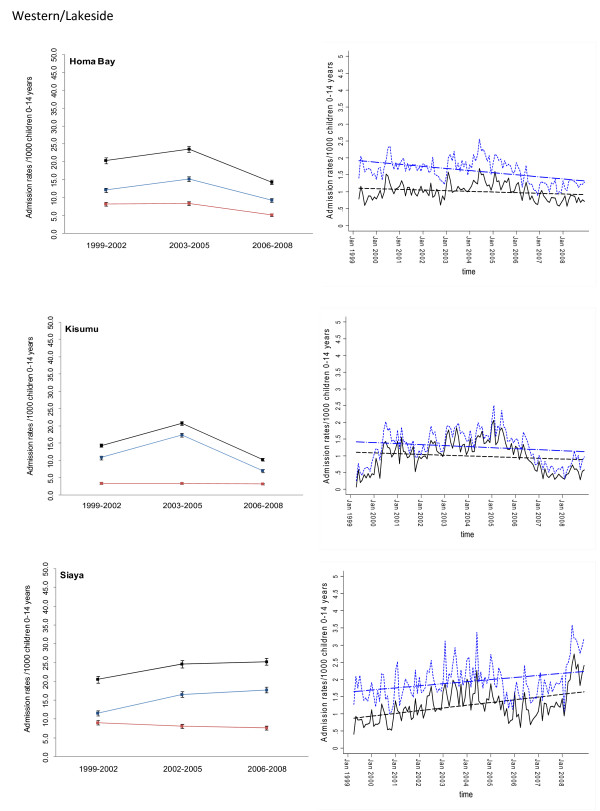
**Plots of paediatric admission data for malaria (blue line) and non-malaria (red line) and all-cause admissions (black line) at three hospitals in Western/Lakeside Region (Homa Bay, Kisumu, Siaya) expressed per 1,000 children aged 0 to 14 years at risk per annum and 95% confidence intervals presented as aggregated data in three time periods: 1999 to 2002, 2003 to 2005 and 2006 to 2008 (Left panels). **Model predictions of all-cause rates controlling for lagged rainfall (dotted blue line) and malaria hospitalization rates controlling for lagged rainfall and non-malaria cases and controlling for autoregressive and moving average effects (solid black line). Fitted lines illustrate the linear trends from model predictions (dashed line) (right panel).

**Figure 4:**
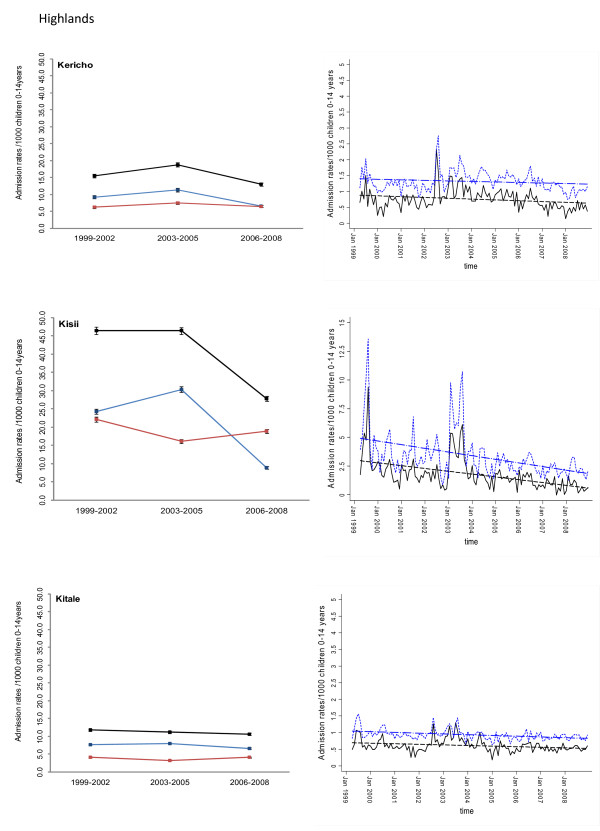
**Plots of paediatric admission data for malaria (blue line) and non-malaria (red line) and all-cause admissions (black line) at three hospitals in the Highlands (Kericho, Kisii, Kitale) expressed per 1,000 children aged 0 to 14 years at risk per annum and 95% confidence intervals presented as aggregated data in three time periods: 1999 to 2002, 2003 to 2005 and 2006 to 2008 (Left panels). **Model predictions of all-cause rates controlling for lagged rainfall (dotted blue line) and malaria hospitalization rates controlling for lagged rainfall and non-malaria cases and controlling for autoregressive and moving average effects (solid black line). Fitted lines illustrate the linear trends from model predictions (dashed line) (right panel).

**Figure 5:**
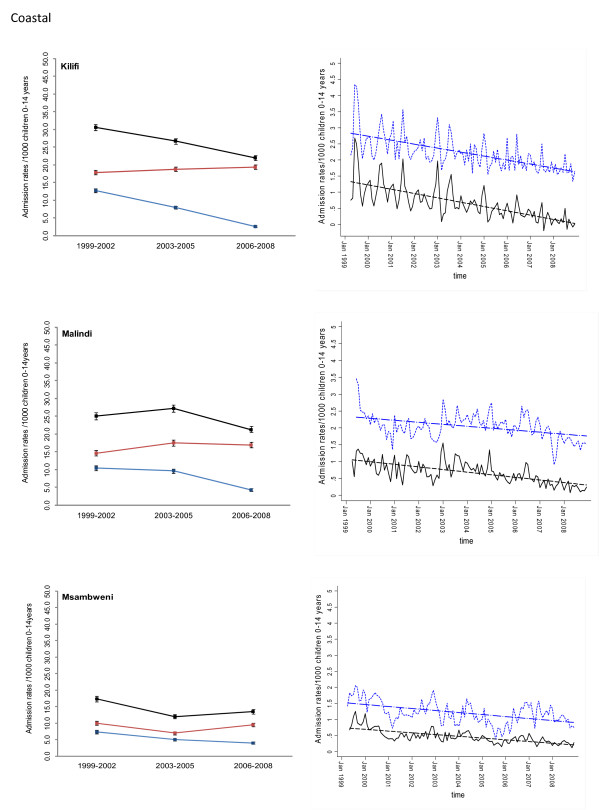
**Plots of paediatric admission data for malaria (blue line) and non-malaria (red line) and all-cause admissions (black line) at three hospitals in on the Kenya coast (Kilifi, Malindi, Msambweni) expressed per 1,000 children aged 0 to 14 years at risk per annum and 95% confidence intervals presented as aggregated data in three time periods: 1999 to 2002, 2003 to 2005 and 2006 to 2008 (Left panels). **Model predictions of all-cause rates controlling for lagged rainfall (dotted blue line) and malaria hospitalization rates controlling for lagged rainfall and non-malaria cases and controlling for autoregressive and moving average effects (solid black line). Fitted lines illustrate the linear trends from model predictions (dashed line) (right panel).

**Figure 6:**
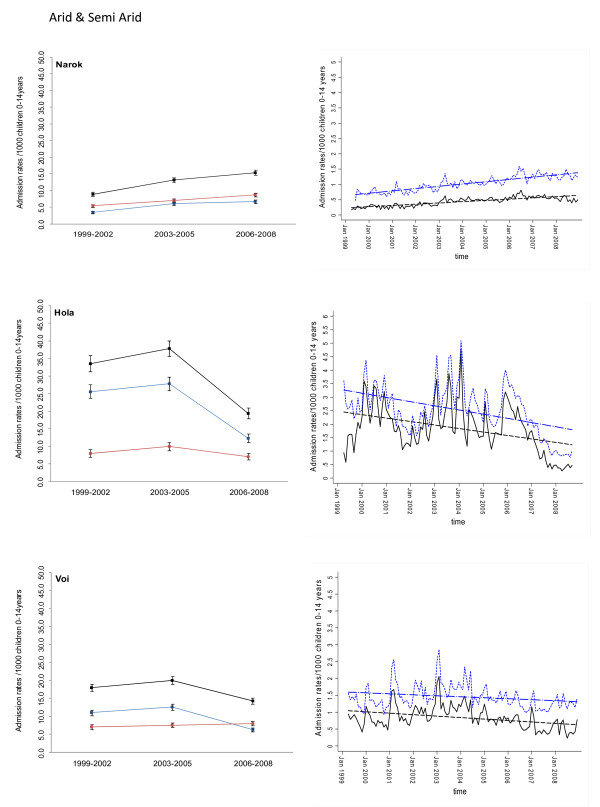
**Plots of paediatric admission data for malaria (blue line) and non-malaria (red line) and all-cause admissions (black line) at three hospitals in the Arid/Semi Arid Region (Narok, Hola, Voi) expressed per 1,000 children aged 0 to 14 years at risk per annum and 95% confidence intervals presented as aggregated data in three time periods: 1999 to 2002, 2003 to 2005 and 2006 to 2008 (Left panels). **Model predictions of all-cause rates controlling for lagged rainfall (dotted blue line) and malaria hospitalization rates controlling for lagged rainfall and non-malaria cases and controlling for autoregressive and moving average effects (solid black line). Fitted lines illustrate the linear trends from model predictions (dashed line) (right panel).

**Figure 7:**
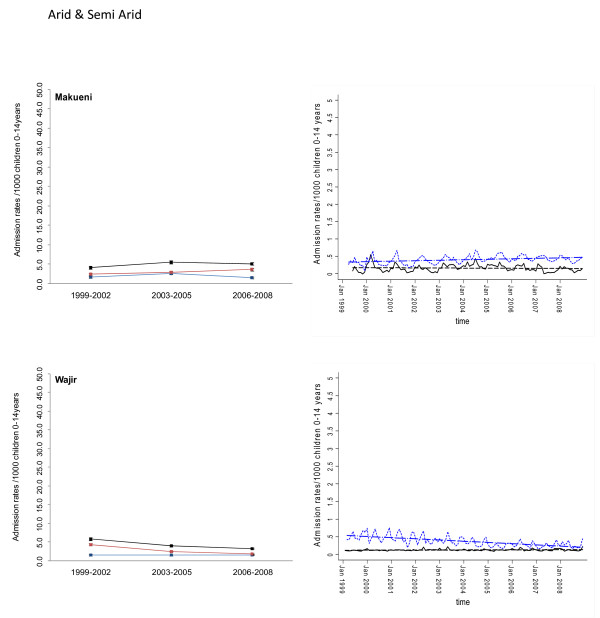
**Plots of paediatric admission data for malaria (blue line) and non-malaria (red line) and all-cause admissions (black line) at two hospitals in the Arid/Semi Arid Region (Makueni, Wajir) expressed per 1,000 children aged 0 to 14 years at risk per annum and 95% confidence intervals presented as aggregated data in three time periods: 1999 to 2002, 2003 to 2005 and 2006 to 2008 (Left panels). **Model predictions of all-cause rates controlling for lagged rainfall (dotted blue line) and malaria hospitalization rates controlling for lagged rainfall and non-malaria cases and controlling for autoregressive and moving average effects (solid black line). Fitted lines illustrate the linear trends from model predictions (dashed line) (right panel).

Between January 1999 and December 2008 information was assembled on 245,810 malaria admissions and 204,128 admissions where a diagnosis of malaria was not made at admission or discharge among children aged less than 15 years of age at the 17 hospitals. Imputing missing values for 45 months using multiple imputation techniques and correcting for the estimated catchment population, rates of malaria admission between 1999 and 2008 varied between hospital sites, with the lowest in the arid areas of Makueni and Wajir and the highest at Kisii in the highlands and Hola located in a predominantly arid area but along the Tana River (Additional file 2: Table SI 1). Overall admission rates were within the range of 1.49 cases per 1,000 children aged 0 to 14 years in Wajir to 21.01 cases in Kisii for malaria admission while non-malaria admission rates were between 2.66 and 19.24 cases per 1,000 children aged 0 to 14 years. Preliminary descriptive data of averaged annualized admission rates for malaria and non-malaria that were congruent with three important control policy time-lines are shown in Figures 2, 3, 4, 5, 6 and 7 (individual time-hospital specific rates provided in Additional file 2: Tables SI 2 and SI 3).

At hospital sites located in the Western/Lakeside region of Kenya malaria admission rates varied little between the three time periods (notably Bungoma) with some evidence of rising rates, remaining elevated in the second (2003 to 2005) and third periods (2006 to 2008) at Busia, Bondo and Siaya (Figures 2 and 3). The exceptions were at Kisumu and Homa Bay where malaria admissions rose in the second time-period and declined in the third. Among hospitals located in the Highlands, both Kericho and Kisii showed a modest decline in malaria admission rates in the third period with much smaller declines at Kitale during the same period (Figure 4). The three hospitals along the coastal region of Kenya show earlier declines in malaria admission rates with lower rates documented in the second period (2003 to 2005) compared to the first time period (1999 to 2002) and further declines in the third period (2006 to 2008) compared to the second period (Figure 5). Hospitals located in areas that are regarded as arid or semi-arid showed variable changes between the three time periods (Figures 6 and 7). At Makeuni and Wajir hospital admission rates with malaria were consistently low across the three time periods. At Narok the rate of malaria admissions doubled between the first and second time periods and remained unchanged during the third time period. Both Hola and Voi however showed similar categorical time-period changes to those in the highlands with no change until the final time period 2006 to 2008 where malaria admission rates more than halved.

Differences were observed in non-malaria admission rates across the three time periods (Figures 2, 3, 4, 5, 6, 7; Additional file 2: Table SI 3). Overall these changes demonstrated a converse pattern to those of malaria admissions with either no changes or marginal declines in rates of non-malaria admission across the three time periods at Busia, Kisumu, Siaya, Kericho, Kitale, Kilifi, Hola, and Voi. At Bungoma, Homa Bay and Wajir rates of non-malaria admission showed an overall decline by 2008. At Bondo, Kisii and Msambweni rates of non-malaria admission declined by 2003 to 2005 compared to the first period (1999 to 2002) but rose again in the last period 2006 to 2008. Rates of non-malaria admission rose across the time periods at Makueni, Narok and marginally at Malindi. The categorical time-defined patterns of paediatric admission for diagnoses that were not malaria were overall not the same as those described for rates of admissions that included malaria as a diagnosis (Figures 2, 3, 4, 5, 6, 7). These variations were used to accommodate general temporal changes in hospital service use during the period in subsequent time-series models.

In general, all-cause admission trends closely matched the temporal patterns of admission observed for malaria diagnoses across almost all study sites (Figures 2, 3, 4, 5, 6, 7). All-cause admission rates varied little between the three time periods at nearly all hospital sites along Lake Victoria and in Western Kenya with the exception of Kisumu and Homa Bay where admissions rose in the second time period and declined in the third (Figures 2 and 3). In the highlands, Kisii showed a significant decline in all-cause admission rates in the third period with much smaller declines at Kericho and Kitale during the same period (Figure 4). In the three hospitals along the Kenyan coast, Kilifi and Kwale showed early declines in all-cause admission rates with lower rates documented in the second period (2003 to 2005) compared to the first time period (1999 to 2002) and further declines in the third period (2006 to 2008) compared to the second period in Kilifi; but remaining unchanged in Kwale (Figure 5). At Malindi declines in all-cause admissions occurred only in the last period. Hospitals located in the arid/semi-arid areas also showed initial rise in the second period followed by a decline in the last period in Hola and Voi; rises in both period two and three in Narok while remaining relatively unchanged in Wajir and Makueni (Figures 6 and 7).

### Adjusted regression analysis of admissions

Different models were explored to test the significance of the temporal trends in the entire 120-month malaria admission rate data series for each hospital separately. Summaries of the best fitting models that adjusted for non-malaria admission rates and/or rainfall at different time-lags are shown for each hospital in Additional file 3. Not surprisingly the amount of rainfall at different monthly lags, between one and four months, prior to an index month significantly influenced the rate of malaria admission in the index month. Only Wajir showed a best fit to rainfall precipitation during the index month rather than a preceding lag. The influence of general service use was explored within the models through the incorporation of monthly non-malaria admission rates or the difference between malaria and non-malaria admission rates. The Table in Additional file 3 shows that for most of the hospitals this parameter of service access/use did show a positive correlation with malaria admissions rates with the exception of the Bondo, Wajir, Voi and Hola time-series. These model forecasts were then used to predict malaria hospitalization rates controlling for lagged rainfall and non-malaria admission rates while controlling for the auto-regressive moving average effects of the data and linear trend lines fitted to estimate the average monthly changes in malaria admission over the 120 time series since January 1999 (Figures 2, 3, 4, 5, 6, 7). Predicted declines in malaria admissions were significant (*P *< 0.05) at Kilifi, Msambweni, Malindi, Kisii, Kericho, Kitale, Bungoma, Homa Bay, Voi and Hola with an average monthly decline of 0.0063 in admission rates since January 1999 (or an average predicted percentage reduction of 49% across all 10 sites by the end of 2008) ranging from 0.0012 drop in admission rates per month at Bungoma to 0.0205 admission rate at Kisii (Figures 2, 3, 4, 5, 6, 7; Table 2) equivalent to a predicted percentage reduction of 25% and 80% respectively by the end of the study period. No significant trends were observed in the time-series at Wajir, Makueni and Kisumu (Figure 3, 7; Table 2). Conversely the trend in malaria admission rates showed a significant rise at Busia, Bondo, Siaya and Narok with an average monthly increase in admission rates of 0.0051 ranging from 0.3400 rise in admission rates per month in Narok (resulting in a predicted rise of 160% by 2008) to 0.0066 in Siaya (corresponding to a predicted rise of 96% by 2008) (Figures 2, 3 and 6; Table 2).

**Table 2:** Summary of trend analysis adjusted for modeled covariates shown in Additional file 3.

Hospital location	Average monthly reduction in malaria admission rates 1999-2008 (95% CI) [*P *value]	Average monthly reduction in total, all-cause admission rates 1999-2008 (95% CI) [*P *value]
**Western/Lakeside**		
**Busia**	0.0051 (0.0039 - 0.0062) [<0.001]	0.0039 (0.0026 - 0.0053) [<0.001]
**Bungoma**	-0.0012 (-0.0023 - -0.0001) [0.037]	-0.0036 (-0.0048 - -0.0024) [<0.001]
**Bondo**	0.0053 (0.0039 - 0.0067) [<0.001]	0.0028 (0.0010 - 0.0045) [0.002]
**Homa Bay**	-0.0016 (-0.0029 - -0.0003) [0.014]	-0.0052 (-0.0068 - -0.0035) [<0.001]
**Kisumu**	-0.0019 (-0.0044 - 0.0005) [0.125]	-0.0025 (-0.0051 - 0.0001) [0.055]
**Siaya**	0.0066 (0.0044 - 0.0088) [<0.001]	0.0050 (0.0023 - 0.0078) [<0.001]
**Highlands**		
**Kericho**	-0.0021 (-0.0038 - -0.0005) [0.013]	-0.0014 (-0.0031 - 0.0003) [0.097]
**Kisii**	-0.0205 (-0.0270 - -0.0140) [<0.001]	-0.0265 (-0.0366 - -0.0164) [<0.001]
**Kitale**	-0.0015 (-0.0025 - -0.0005) [<0.001]	-0.0019 (-0.27 - -0.11) [<0.001]
**Coastal**		
**Kilifi**	-0.0112 (-0.0132 - -0.0091) [<0.001]	-0.0103 (-0.0126 - -0.0080) [<0.001]
**Malindi**	-0.0064 (-0.0078 - -0.0051) [<0.001]	-0.0049 (-0.0069 - -0.0029) [<0.001]
**Msambweni**	-0.0045 (-0.0053 - -0.0037) [<0.001]	-0.0052 (-0.0069 - -0.0036) [<0.001]
**Arid & semi-arid**		
**Narok**	0.0034 (0.0030 - 0.0039) [<0.001]	0.0063 (0.0056 - 0.0069) [<0.001]
**Hola**	-0.0107 (-0.0155 - -0.0060) [<0.001]	-1.30 (-1.77 - -0.82) [<0.001]
**Voi**	-0.0036 (-0.0053 - -0.0019) [<0.001]	-0.0024 (-0.0043 - -0.0006) [0.011]
**Makueni**	-0.0002 (-0.0008 - 0.0003) [0.424]	0.0012 (0.0006 - 0.0017) [<0.001]
**Wajir**	0.0001 (0.0000 - 0.0003) [0.066]	-0.29 (-0.0036 - -0.0023) [<0.001]

The monthly change represents rates expressed per 1,000 children aged 0 to14 years.

To examine the trends in all-cause admission rates similar model approaches were developed and their resulting trends are shown for comparative purposes in Figures 2, 3, 4, 5, 6, 7 and the proportional changes in monthly all-cause admission rates through to the end of 2008 are shown in Table 2.

## Discussion

We have examined the temporal patterns of paediatric malaria admissions at 17 hospitals across the diverse malaria transmission ecology of Kenya between January 1999 and December 2008. At two hospital sites located in arid areas (Makueni and Wajir) hospitalization from malaria (and non-malaria) was extremely low and showed no obvious changes during the observation period. At 10 of the remaining 15 sites reductions in malaria admission rates were observed. These were significant after adjusting for rainfall and general hospital service use. The overall reduction in the paediatric malaria admission rate was on average 0.0063 per month since January 1999 by the end of the 120-month time-series. These sites included the three areas located along the Kenyan coast, three areas in the highlands, two semi-arid areas: Voi and Hola (along the Tana river), and two sites located in the west of Kenya: Homa Bay along the shores of lake Victoria and Bungoma bordering Uganda. Homa Bay and Bungoma, however, showed small proportional reductions in monthly malaria admissions over the 120-month time-series and not as marked as the remaining eight sites showing overall declines in malaria admissions (Table 2; Figures 2, 3, 4, 5, 6, 7). Despite statistical significance of the time-series adjusted analysis of malaria admissions, largest reductions with the clearest trends were seen at Kisii, Kilifi, Malindi and Msambweni. Reductions at the other six hospital sites were less marked and subject to large within-series fluctuations in admission rates. At Kericho an exceptional peak in malaria admissions in 2002 was an anomaly (Figure 4); the large initial peak in malaria admissions at Bungoma contributed to the perceived modeled decline in risk with time (Figure 2); and at Kitale, Homa Bay, Voi and Hola, malaria admissions rose from 1999 to 2003 through 2005, but were then off-set against declines in admission rates between 2006 and 2008 (Figures 3, 4 and 6). This was also true of Kisumu that showed no overall significant rise or decline across the combined 120-month time series (Figure 4).

A more consistent pattern of increasing adjusted malaria admission rates over the observation period was observed at Busia, Siaya and Bondo district hospitals where the rise in malaria admission rates was between 0.51% and 0.66% per month by the end of the time-series in December 2008 (Table 2). Following a rise in admissions from January 1999, Siaya exhibited a drop in malaria admission rates after January 2005 but rose again through 2008 (Figure 3).

The analysis assumed that the trend component was linear; meaning that the level of the predicted series increases by a constant amount each time and this is valid across the majority of the data series. However we acknowledge that in certain instances, the series do not exhibit a constant time trend and are extremely variable moving up or down over the course of a few years as appears to be the case in Hola and Kisumu. An additional caveat is that we have assumed a constant annualized growth in population size using inter-censal estimates from 1989 to 1999; it was not possible to derive a more precise and temporally consistent growth rate in the absence of the 2009 national census and therefore we cannot be certain of changing population size and structures.

It is equally important to recognize the 17 hospitals selected here were those that could provide enough information over a long enough time period to evaluate trends in paediatic admission and only one hospital had consistently monitored the presentation of malaria with microscopy and detailed clinical examination of all admissions (Kilifi District Hospital). Thus the majority of the data analyzed here are not based upon parasitological confirmed cases of malaria. These data were often not available and of variable quality and accuracy [32,49]. Changes in clinical diagnosis of malaria with time may have contributed a systematic, non-random error in the data series reflecting periods of perceived *epidemics *in the highlands or perceived *absence *of malaria following wide-spread reporting of malaria declines in Kenya by the media. However, in the past malaria has been the predominant clinical and parasitological confirmed diagnosis made at Kilifi district hospital where rigorous screening of paediatric admissions prevailed throughout the period of observation [16] and accounted for 27% of all diagnoses made in children aged less than 15 years in the 1999 to 2008 series included here. Assuming malaria is a significant contributor to admissions to hospital among children aged less than 15 years, it is hard to clinically define, and assuming all else being equal after adjusting for increasing populations in the catchment areas and inter-annual changes in seasonal rainfall, one would anticipate a decline in *all-cause *paediatric admission rates from 1999. This was the rationale behind the large-scale, community randomized trials of insecticide treated nets during the 1990s where all-cause childhood mortality was the principal clinical outcome in Phase III trials [50]. Under these assumptions the only significant declines in all-cause admission rates were seen to any great extent at Homa Bay, Kisii, Kilifi, Malindi, Msambweni and Hola with an overall reduction in admission rates of 0.0050 or more per month since January 1999. These observations would suggest that apparent reductions observed for malaria admissions in these six sites are true reductions and was not an artifact of misdiagnosis.

We have applied a rigorous set of time-series models to an extended series of hospital admissions in an attempt to provide some externality to earlier observations of declining malaria admissions at one hospital (Kilifi [16]) and at three hospitals (Kilifi, Msambweni and Malindi [15]) on the Kenyan coast using more covariates against standardized populations. Providing more data from more sites is important to validate and test general patterns of health transition. In doing so we have shown that, despite the difficulties in interpreting time-series hospital data, patterns observed on the Kenyan coast are not replicable across the country. In general terms malaria admissions along the coast had begun to decline before the wide-scale introduction of prevention strategies using ITN and the introduction of new, effective therapeutics into the government health service. In other areas of Kenya malaria admission rates had risen from 1999 to 2006 but showed a variable decline after 2006. Whether this was due to scaled intervention coverage or not remains uncertain and would require a much longer post-2006 time series to be able to rule out short-term periodicity in malaria transmission. Several sites showed plausible evidence of increasing malaria admission risks across the entire time-series and thus do not support the hypothesis of health impact following scaled ITN delivery or the switch from failing to effective treatment. Interestingly there was some suggestion that areas with more pronounced declines in malaria admission rates were areas with lower intensity parasite transmission at the end of the surveillance period compared to areas with higher ending transmission intensity that showed no change or a rise in cases of malaria during the interval. While our study did not examine changing infection prevalence with time, theory does suggest that the clinical impact of interventions targeted at reducing parasite exposure may be more immediate in areas of lower transmission intensity [51,52]. With a higher baseline, starting transmission intensity the impact on parasite exposure, and subsequent disease outcomes will be slower and will initially transition through a period of higher burden as older children become increasingly at risk due to changing age-specific immune profiles [51,52] and will only change when transmission intensity has reached an ill-defined point. We are exploring this hypothesis separately with better data on infection prevalence transitions and changing intervention coverage in selected sites included here.

Because Kenya has made significant progress at a national level on changing its drug policy to support effective case-management [43] and represents one of only a few countries in Africa that have massively increased ITN coverage since 2006 [1] we had anticipated a replication of observations made on the Kenyan coast [15,16] at other hospital sites across the country. This was not the case and our data are an important reminder that all is not equal and single, spatially constrained observations within a country may be misleading. In this regard, it is important to highlight the fact that efforts geared towards improved intervention coverage may not have been implemented uniformly countrywide.

## Conclusion

A country-wide assessment of trends in malaria hospitalizations indicates that all is not equal, important variations exist in the temporal pattern of malaria admissions between sites and these differences require more detailed investigation to understand what is required to promote a clinical transition across Africa.

## Abbreviations

ACT: Artemisinin-based combination therapies; AIC: Akaike Information Criterion; AR: autoregressive; ARMAX: Autoregressive moving average model with exogenous inputs (X); BIC: Bayesian Information Criterion; EA: Enumeration Areas; EVI: Enhanced Vegetation Index; ITN: insecticide treated nets; MA: moving average; *Pf *PR_2-10 _: *Plasmodium falciparum *Parasite Prevalence in children aged 2 up to 10 years; SP: sulphadoxine-pyrimethamine.

## Competing interests

Some of the data have been presented at a Novartis sponsored symposium at the American Society of Tropical Medicine and Hygiene, New Orleans, LA, USA, December 2008. The sponsors of the presentation have had no role in the publication of the final results. We declare no further competing interests.

## Authors' contributions

EAO was responsible for study design, data cleaning, analysis, interpretation and production of the final manuscript. VAA contributed to the development of geo-statistical models used to define hospital catchment areas and contributed to the final manuscript. AMN provided modelled interpolated temporal estimations of *Pf *PR_2-10 _in each of the catchment areas and contributed to the final manuscript. JJM assisted in the primary assembly of the hospital data at each of the 17 hospitals. EJ contributed to supervision of data collection, provision of general policy framework and production of final manuscript. RWS was responsible for overall scientific management, analysis, interpretation and preparation of the final manuscript.

## Pre-publication history

The pre-publication history for this paper can be accessed here:

http://www.biomedcentral.com/1741-7015/7/75/prepub

## Supplementary Material

Additional file 1 Defining the Health Facility Catchment Population. Click here for file

Additional file 2 Supplementary tables. Click here for file

Additional file 3 ARMAX regression model coefficients of the monthly malaria incidence (1999-2008) on rainfall and non-malaria case incidence for each hospital site. Click here for file
