# Supplementary material for: Malaria paediatric hospitalization between 1999 and 2008 across Kenya
Source: BMC Med. 2009 Dec 9;7:75. doi: 10.1186/1741-7015-7-75 (PMC2802588; doi:10.1186/1741-7015-7-75)
Supplement: Additional file 1 — Defining the Health Facility Catchment Population. [file 1741-7015-7-75-S1.DOC]

# Supplementary Infromation 1: *Defining the Health Facility Catchment Population*

For all in-patients, data on the village and/or sub-location (the lowest administrative unit) of residence are routinely collected and entered into the registers at the hospitals. To account for possible variation in hospital usage by season data for all patients that were admitted to the 17 hospitals in March, June and October 2007 were assembled, excluding patients with missing information on residence. All villages or sub-locations were then geo-located using a combination of national databases of villages and other settlements [1]. Where villages could not be positioned from these sources, centroids of enumeration area (EA) polygons, the smallest population unit used in the 1999 Kenya national census [2] and often an equivalent of a small village was used. The size of an EA was extremely variable ranging from 2km^2^ to 295km^2^ across the study districts.

Data points were then displayed in ARCGIS 9.2 (ESRI Inc, Redland, CA, USA) and the distance from each village to the district hospital was calculated. A plot of the distance against the number of people admitted to a hospital was constructed. The distance at which the utilization began to decline was identified for each hospital and using a radius equivalent to this threshold distance, a circular buffer was generated around the hospital. Then a convex hull polygon, connecting the outer extent of the furthest villages from which 90% of in-patients at the hospital were admitted, was generated. The circular buffer and the convex hull polygon were then intersected and those portions of the buffer or the polygon from which there were no patients visiting the hospital were erased. The remaining areas around the hospitals derived through the combination of the circular buffer and the convex hull were then visually inspected and minimal subjective alterations incorporated to optimize catchment areas using, as the basis for modification, knowledge on road networks and physical barriers, the actual data points and *a priori* information. The boundaries of these areas were then smoothed and were designated as the hospital’s catchment area and were used for all subsequent analysis. These catchment areas were overlaid on a high-resolution population map [3] projected to each year of survey to derive the hospital catchment population for the year of study.

**Table: The table below shows the characteristics used to define catchment population in Kenya and indicates the specificity of catchment documentation, the total number of people (population census 1999) and EA polygons in defined catchment zones**

| **Hospital Location** | **Total admissions captured for the 3 months** | **Number of paediatric admissions used to define catchment** | **Total persons Mapped** | **Number of people mapped at EA level** | **Number of people mapped at Sub-location level** | **Total persons Not Mapped** | **Total number of EA Polygons mapped** | **Total Sub-location Polygons mapped** | **1999 Population in catchment** | **Total Number of EA polygons in District** | **Total Number of EA polygons in Final Catchment** | **Total Number of Sub-location polygons in Final Catchment** |
| --- | --- | --- | --- | --- | --- | --- | --- | --- | --- | --- | --- | --- |
| **Western/ Lakeside** |  |  |  |  |  |  |  |  |  |  |  |  |
| Busia | 640 | 365 | 365 | 365 | 0 | 0 | 115 | 0 | 369,552 | 754 | 780 |  |
| Bungoma | 655 | 477 | 449 | 449 | 0 | 28 | 214 | 0 | 569,953 | 1,621 | 1,066 |  |
| Bondo | 326 | 230 | 244 | 244 | 0 | 0 | 55 | 0 | 153,864 | 672 | 451 |  |
| Homa Bay | 514 | 234 | 224 | 216 | 8 | 10 | 80 | 3 | 253,496 | 719 | 564 | 4 |
| Kisumu | 547 | 284 | 283 | 283 | 0 | 1 | 117 | 0 | 541,002 | 1,289 | 1,300 |  |
| Siaya | 662 | 644 | 642 | 642 | 0 | 2 | 86 | 0 | 243,149 | 1,371 | 714 |  |
| **Highlands** |  |  |  |  |  |  |  |  |  |  |  |  |
| Kericho | 700 | 232 | 203 | 0 | 203 | 29 | 0 | 68 | 406,491 | 656 |  | 93 |
| Kisii | 1362 | 302 | 290 | 272 | 18 | 12 | 143 | 14 | 405,331 | 1,069 | 668 | 13 |
| Kitale | 1225 | 1387 | 1292 | 1266 | 26 | 95 | 243 | 2 | 765,943 | 909 | 992 | 20 |
| **Coastal** |  |  |  |  |  |  |  |  |  |  |  |  |
| Kilifi | 414 | 617 | 569 | 569 | 0 | 48 | 158 | 0 | 338,112 | 1,079 | 670 |  |
| Malindi | 519 | 364 | 349 | 349 | 0 | 15 | 71 | 0 | 201,049 | 566 | 407 |  |
| Msambweni | 469 | 214 | 204 | 204 | 0 | 10 | 81 | 0 | 241,647 | 812 | 434 |  |
| **Arid and Semi-Arid** |  |  |  |  |  |  |  |  |  |  |  |  |
| Narok | 466 | 433 | 433 | 433 | 0 | 0 | 63 | 0 | 167,519 | 861 | 383 |  |
| Hola | 125 | 112 | 112 | 112 | 0 | 112 | 37 | 0 | 52,622 | 503 | 128 |  |
| Voi | 243 | 219 | 198 | 198 | 0 | 21 | 84 | 0 | 159,230 | 619 | 406 |  |
| Makueni | 188 | 202 | 200 | 200 | 0 | 2 | 139 | 0 | 293,997 | 555 | 603 |  |
| Wajir | 271 | 245 | 239 | 239 | 0 | 6 | 53 | 0 | 287,617 | 409 | 379 |  |

Map of seventeen district showing hospital catchments (Enumeration Area- EA, 1999). Each map shows final defined catchment zone for each hospital site highlighted in orange. The maps show EA within each district. Those shaded in green represent those EAs with admissions and the numbers indicate the number of admissions from each EA .

**I. Western/ Lakeside region:**

**
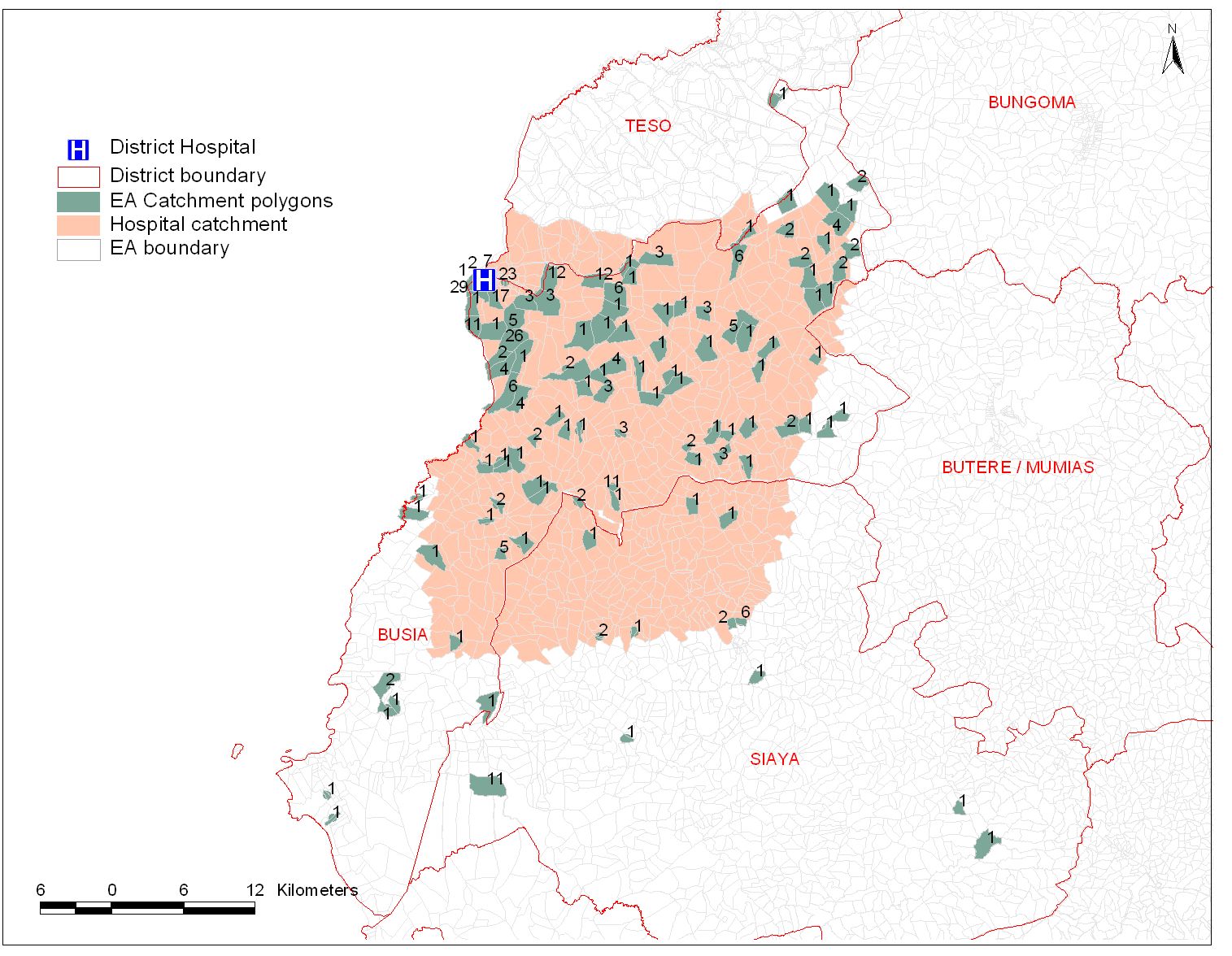

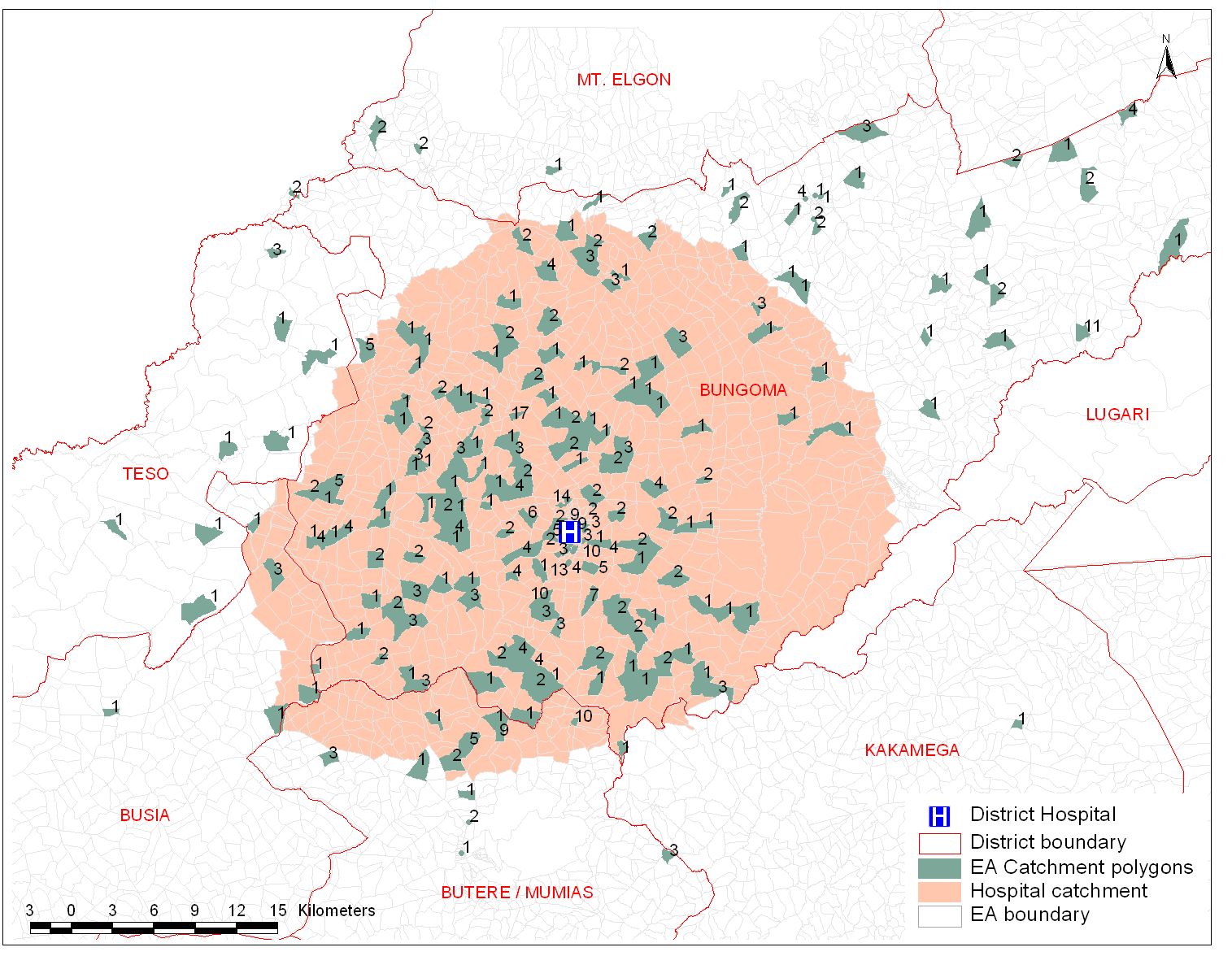
**

1. **Busia b) Bungoma**


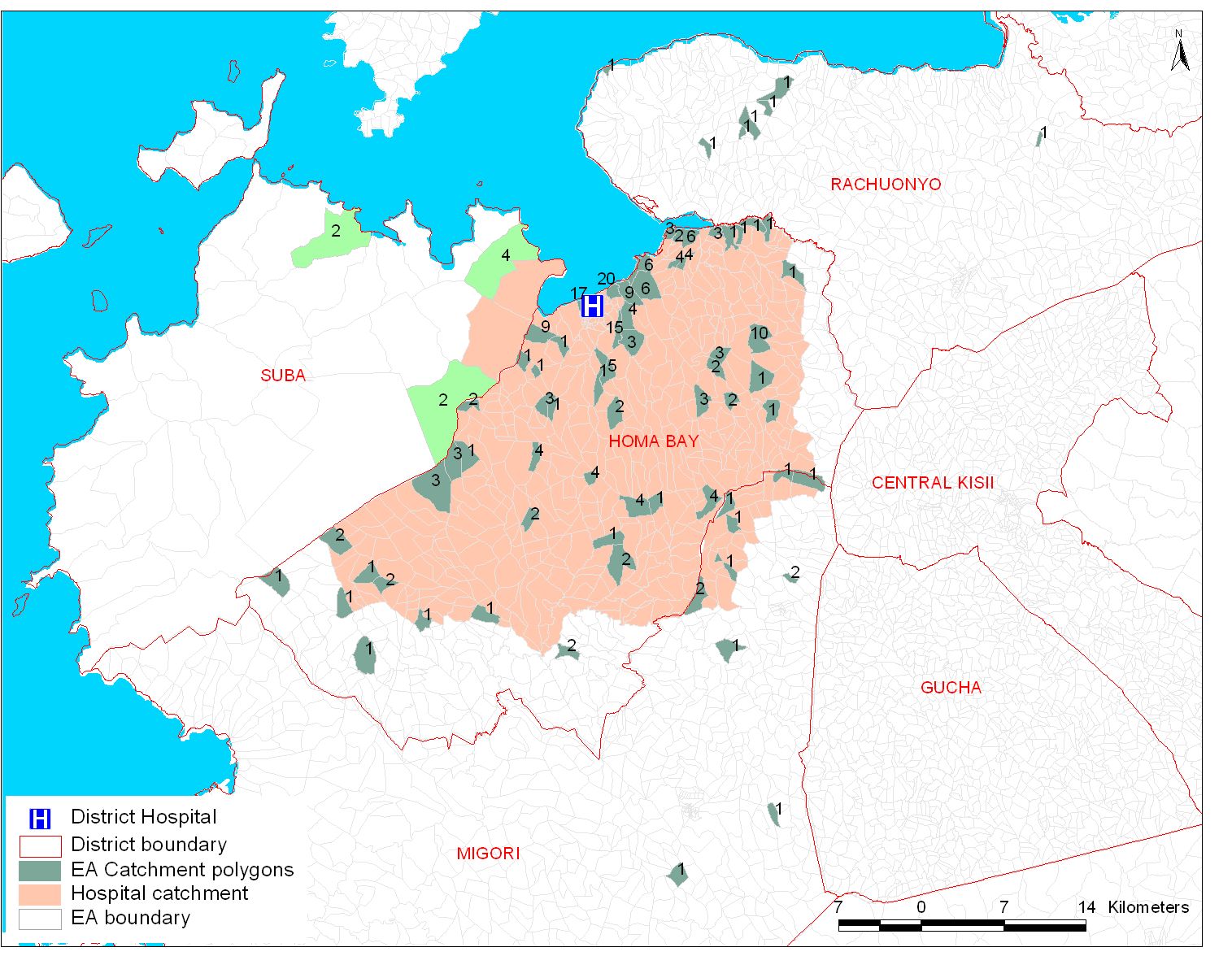

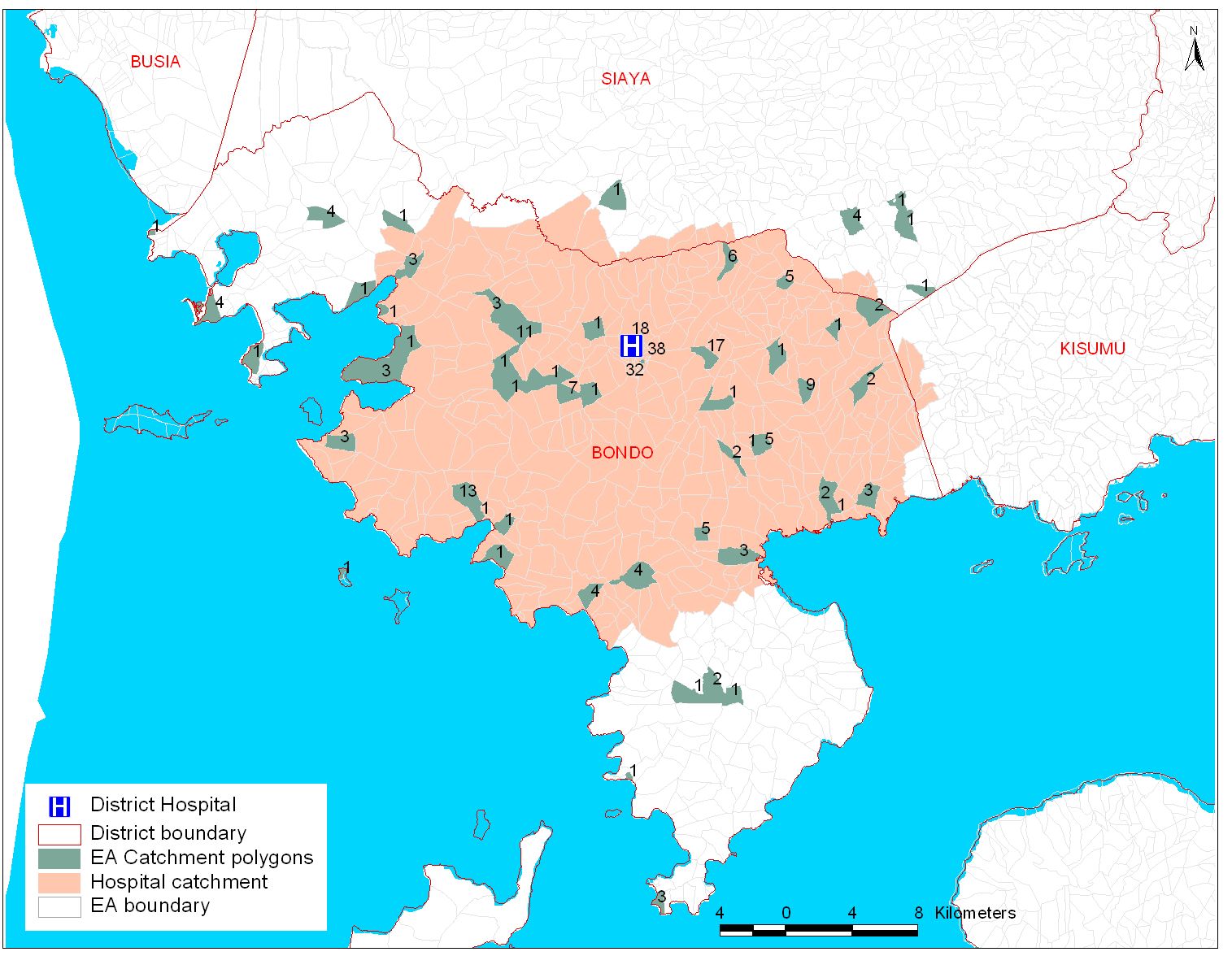
 **I. Western/Lakeside region:**

**c) Bondo d) Homa Bay**


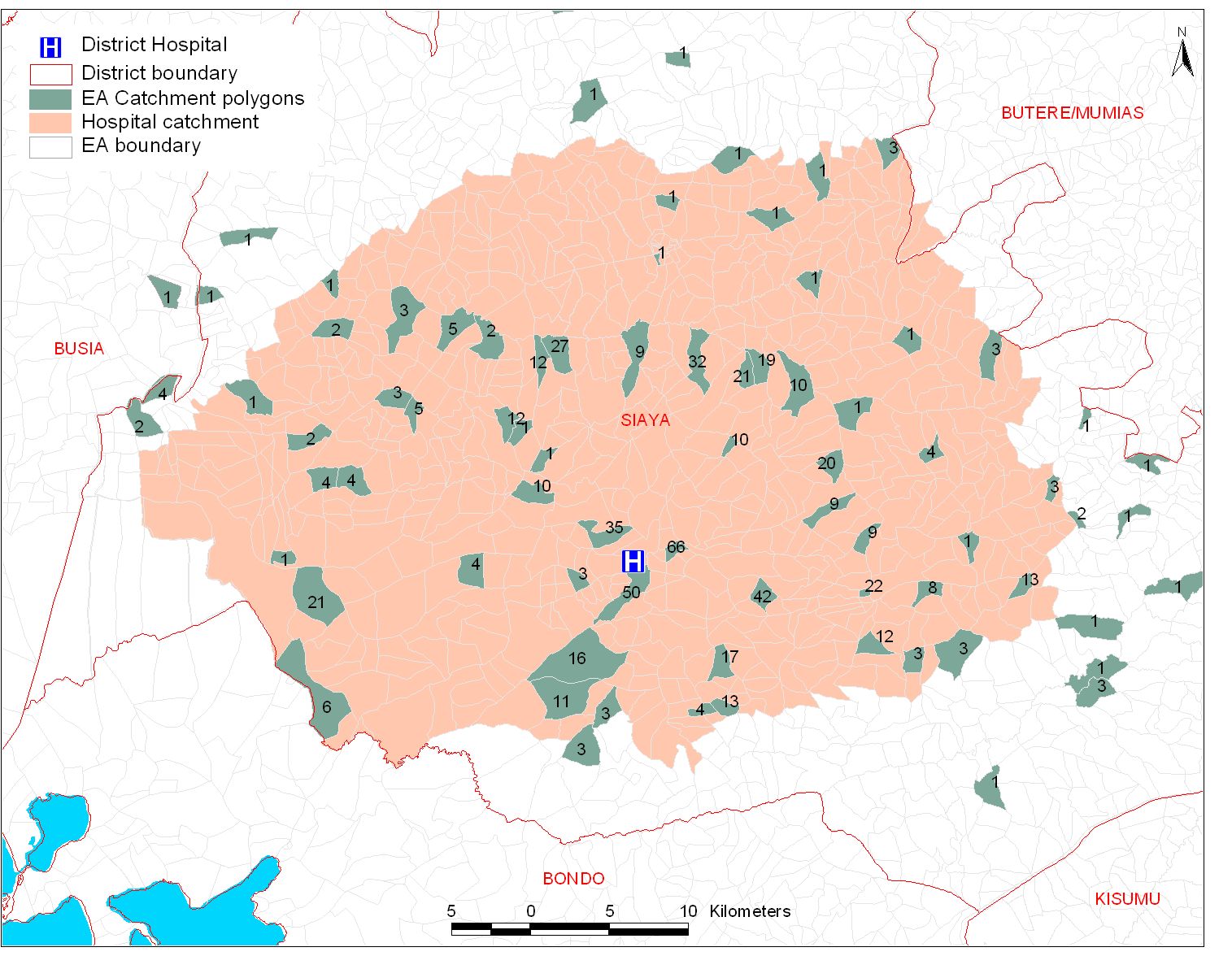
**I. Western Lakeside region:**

**
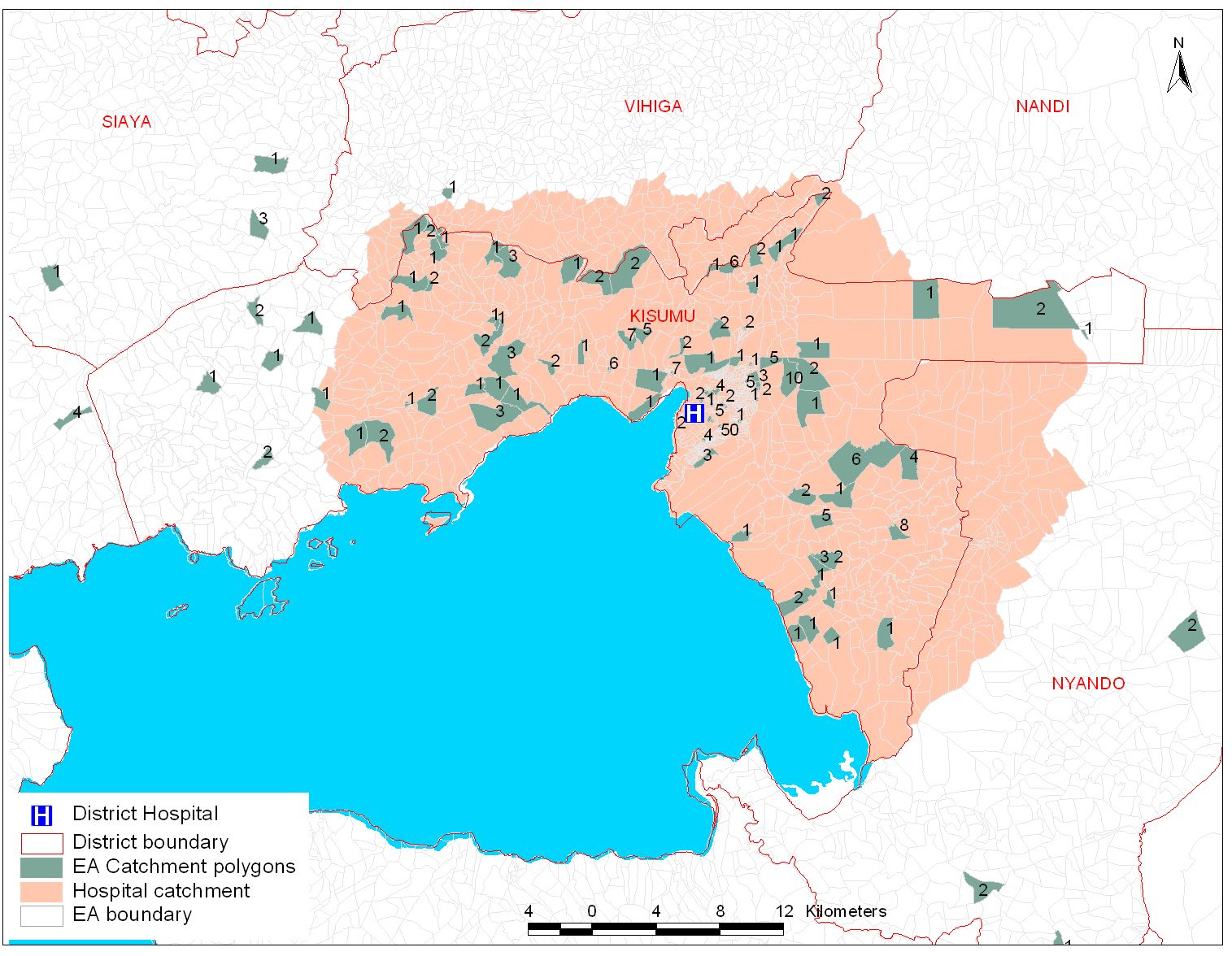
**

1. **Kisumu f) Siaya**


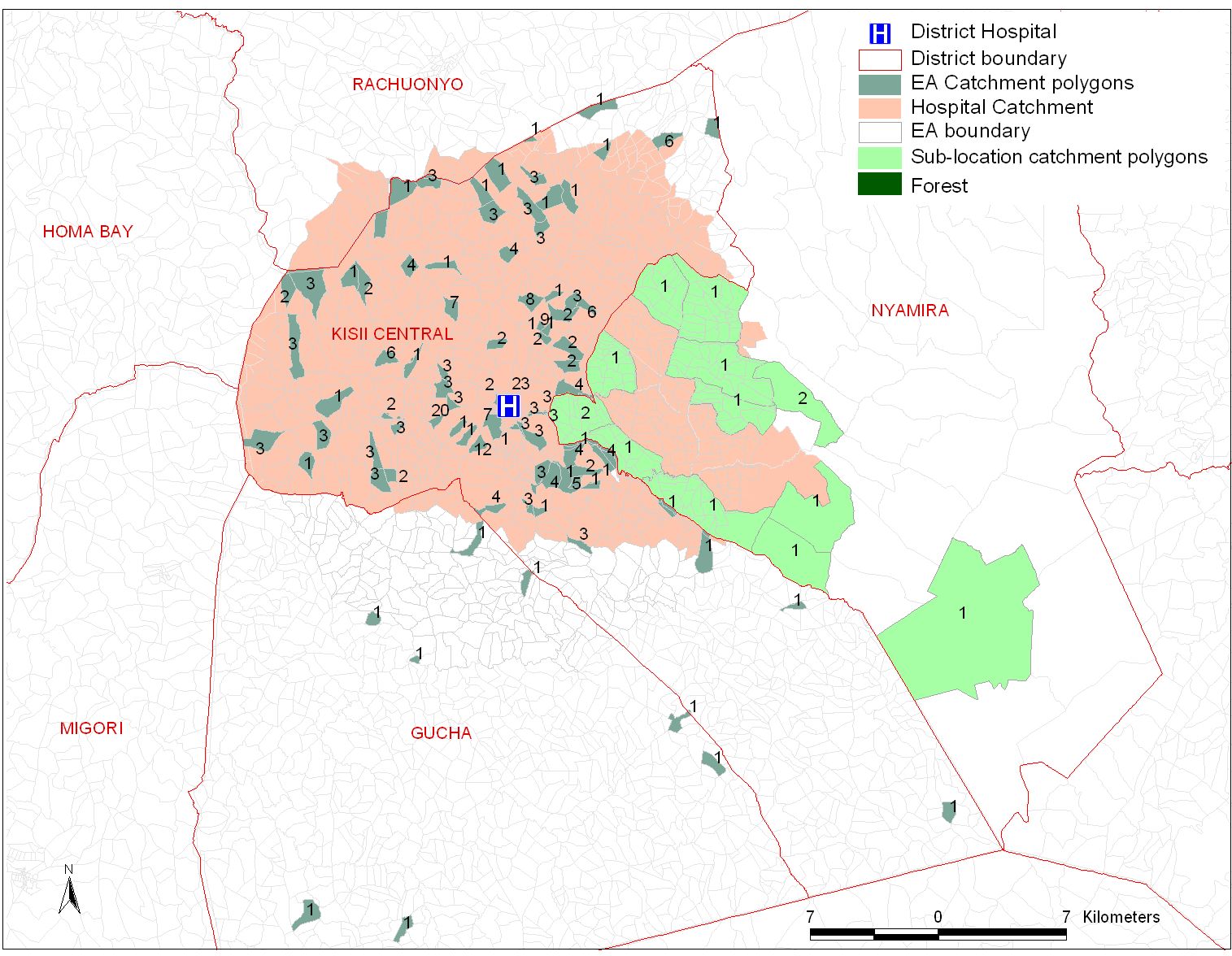

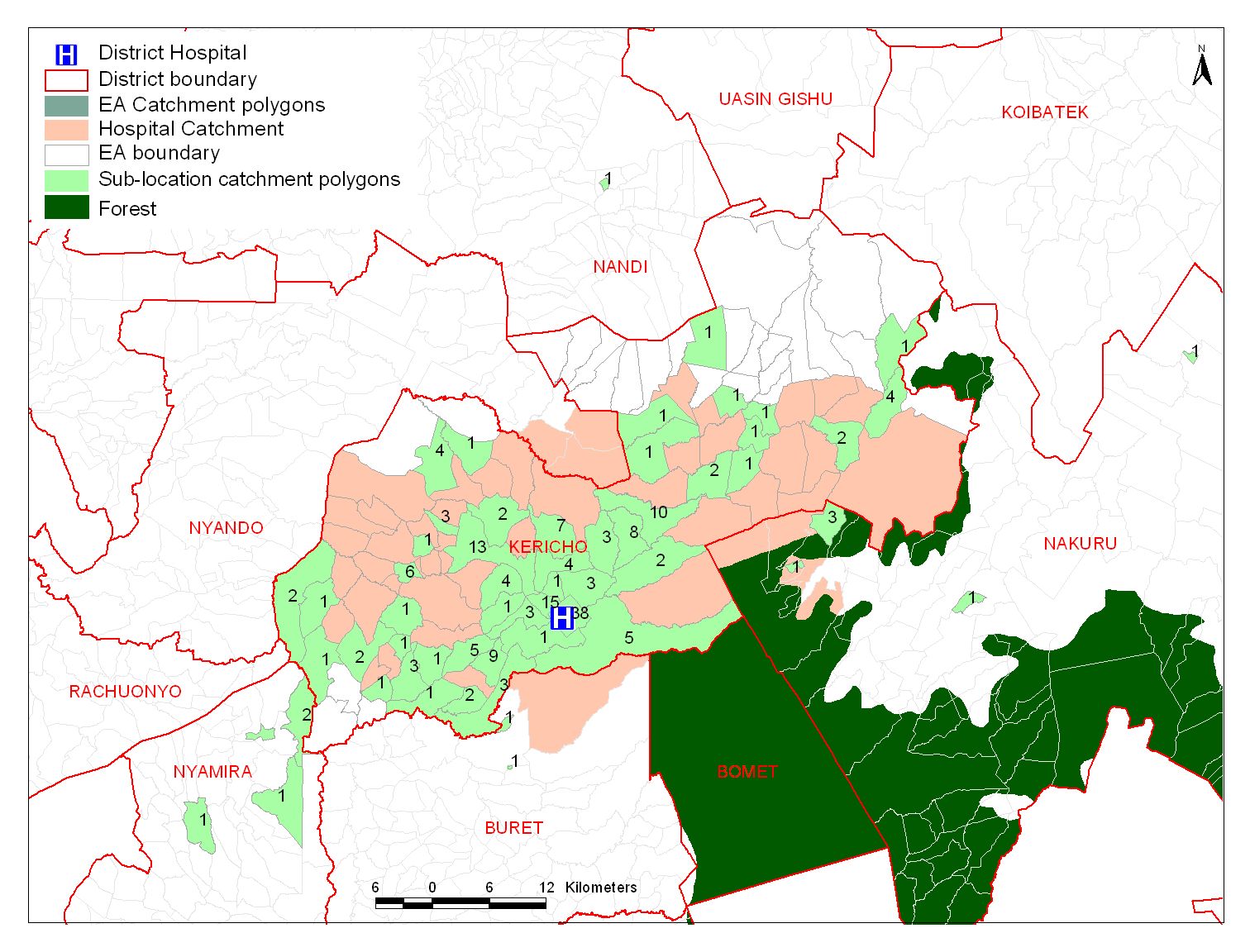
**II Highlands:**

**a) Kericho b) Kisii**

**II Highlands:**

**
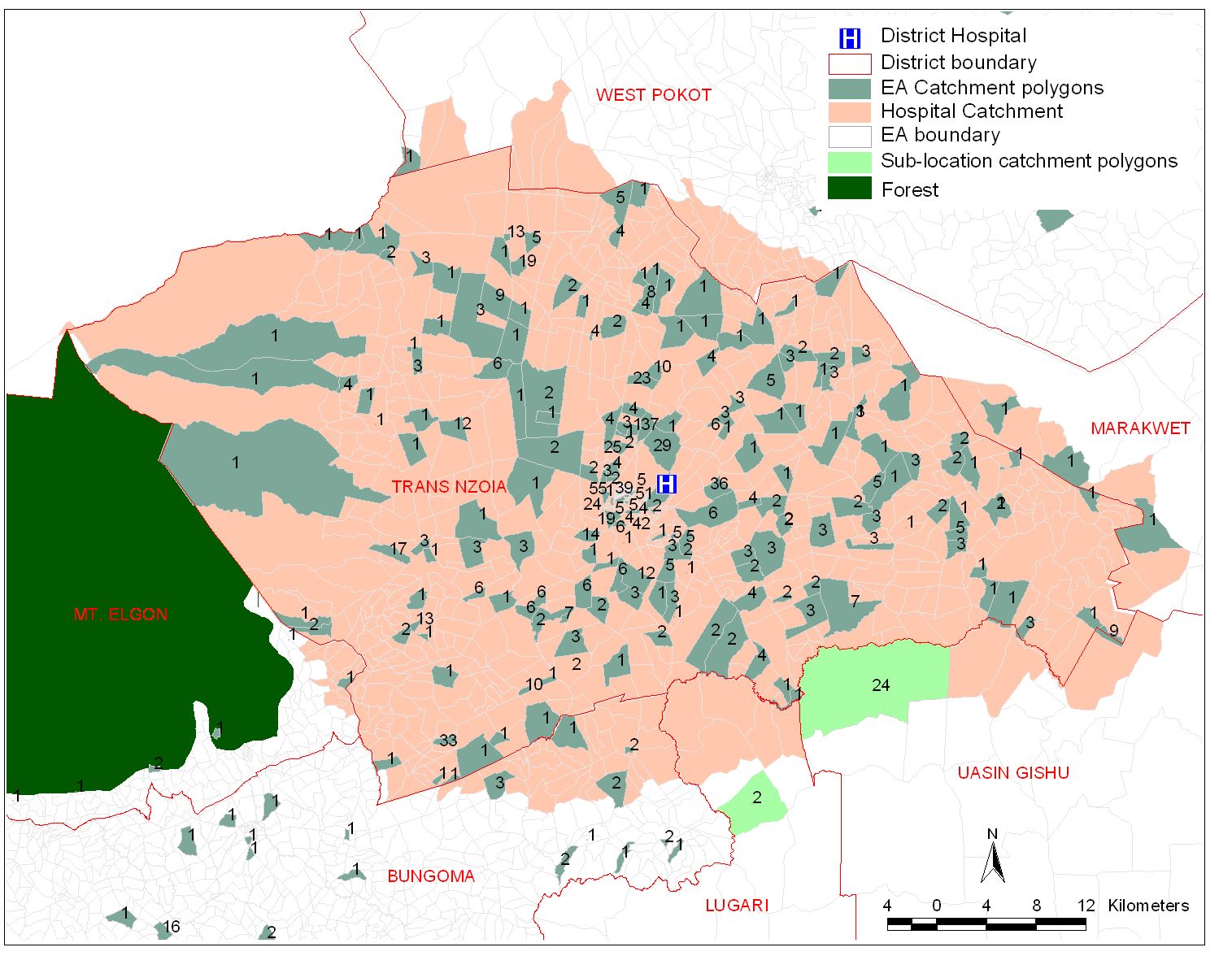
**

**c) Kitale (Trans Nzoia District)**

**III. Coastal region:**

**
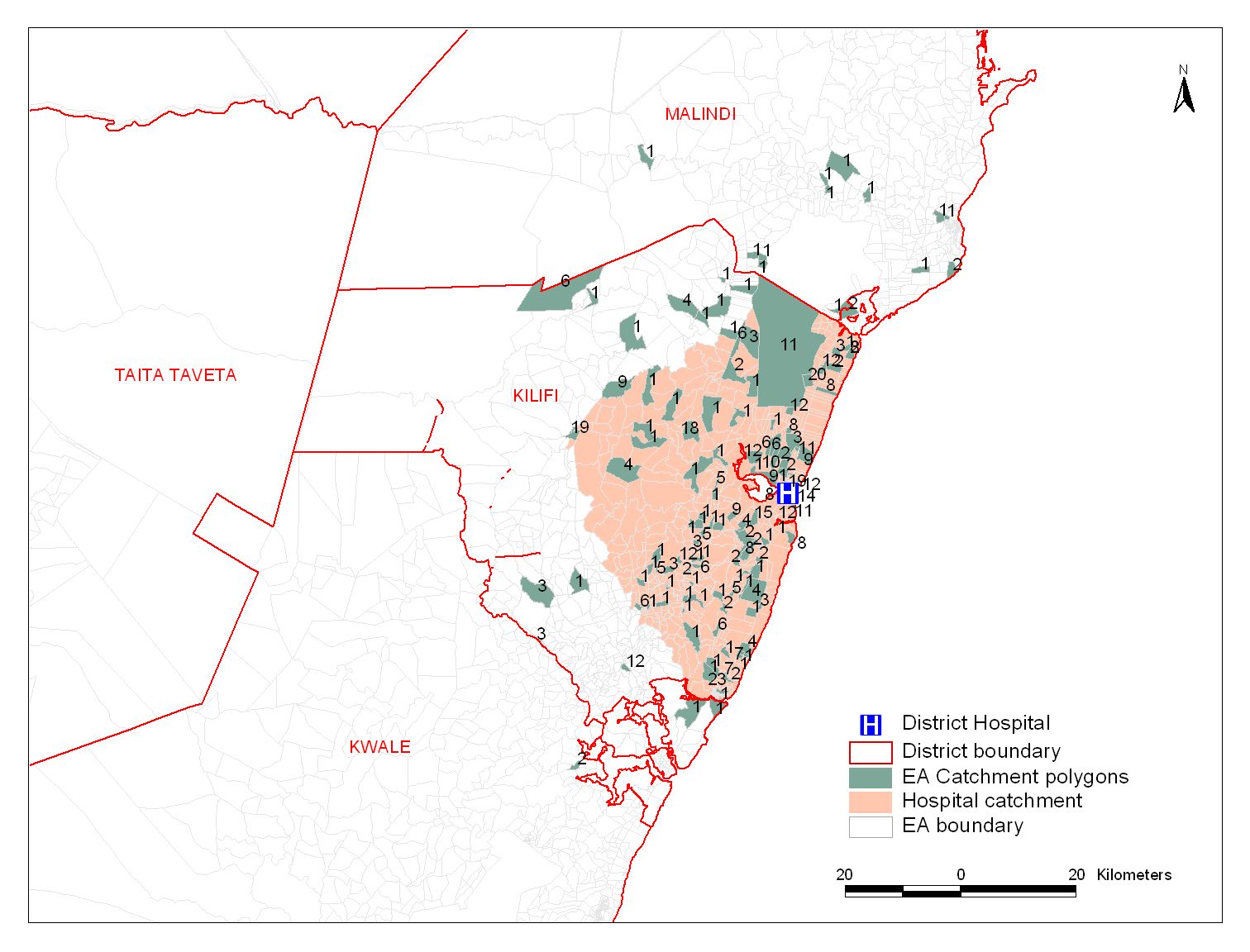

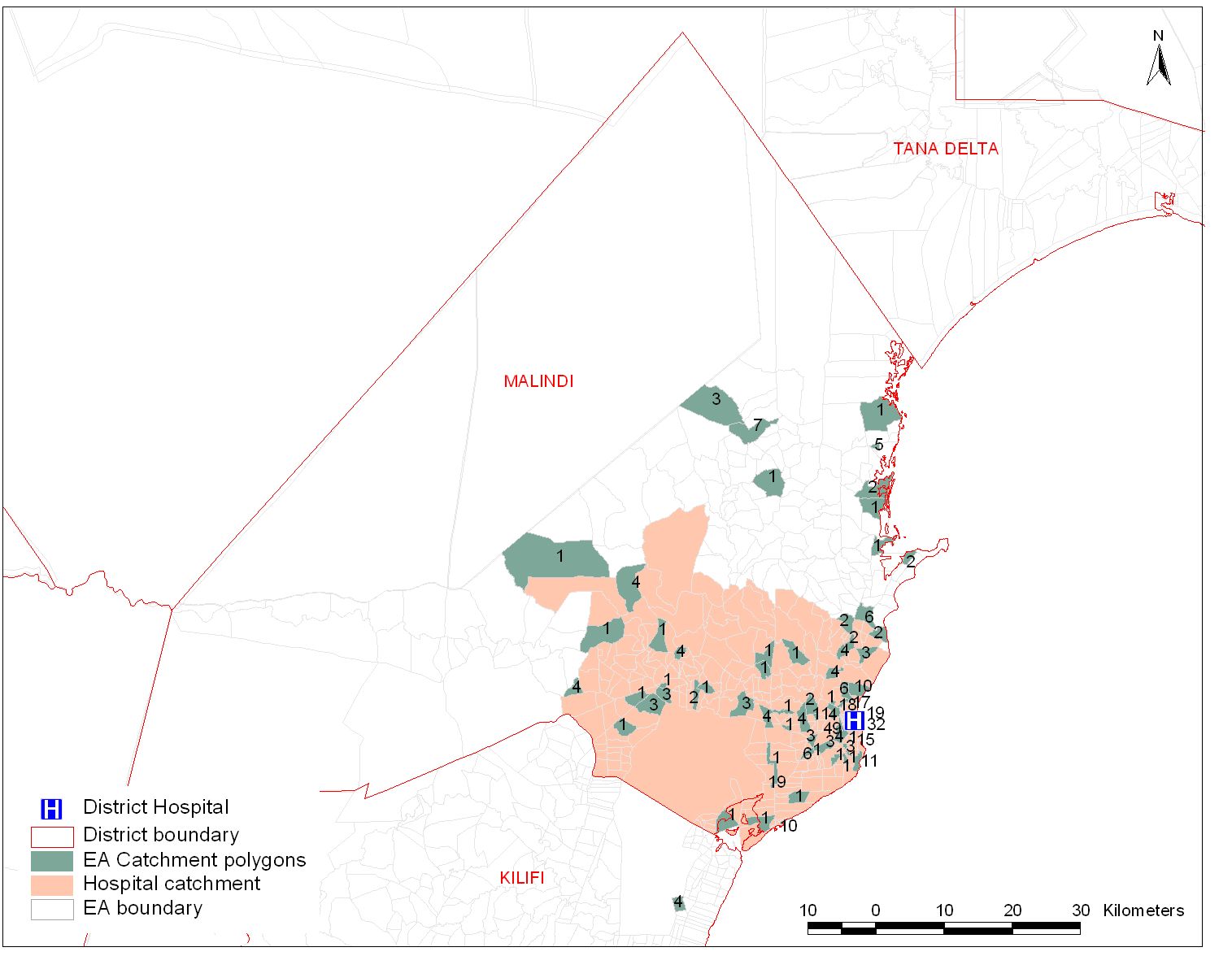
**

**a) Kilifi b) Malindi**

**IV. Coastal region:**

**
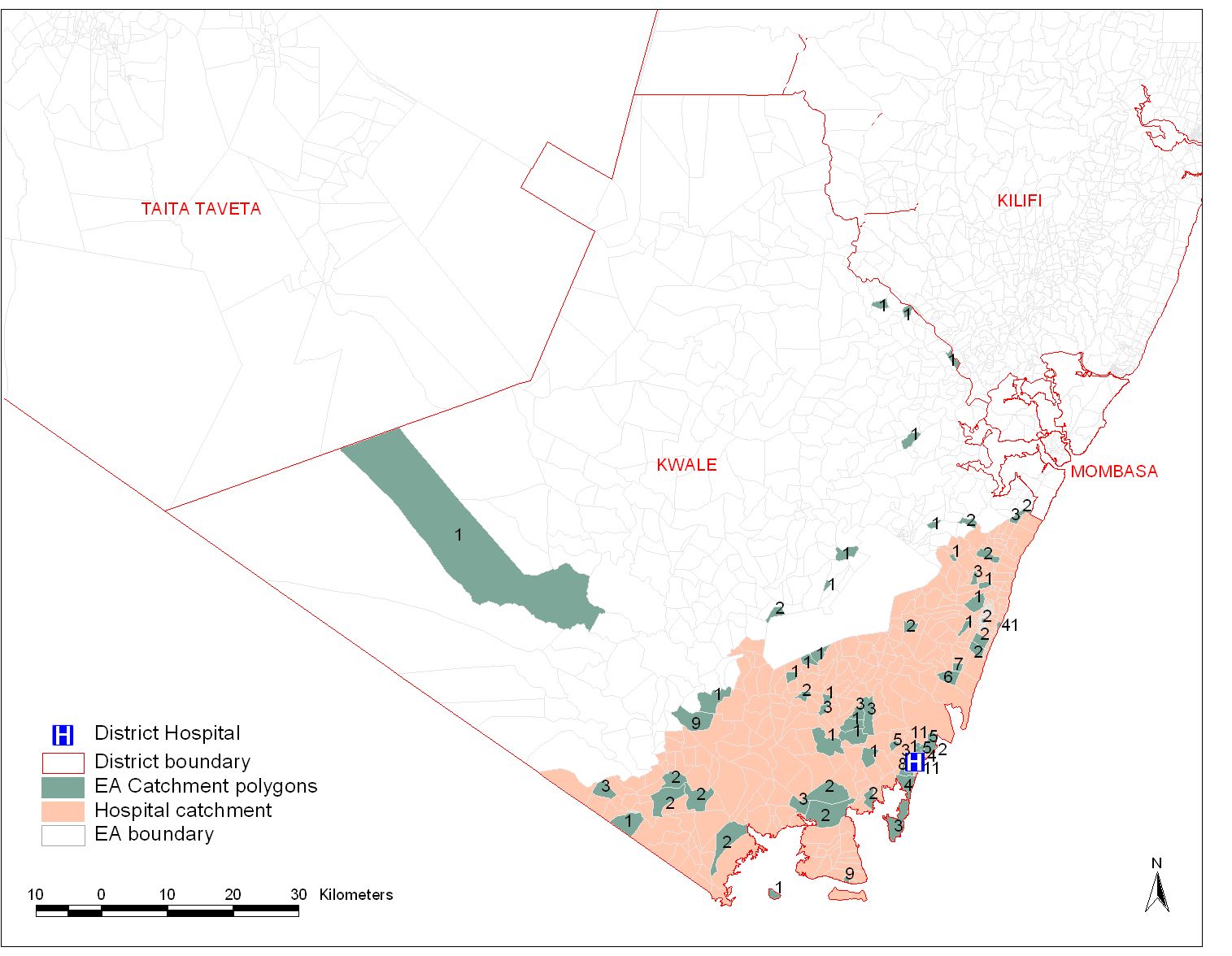
**

**c) Msambweni**

**IV. Arid and Semi-Arid areas:**

**
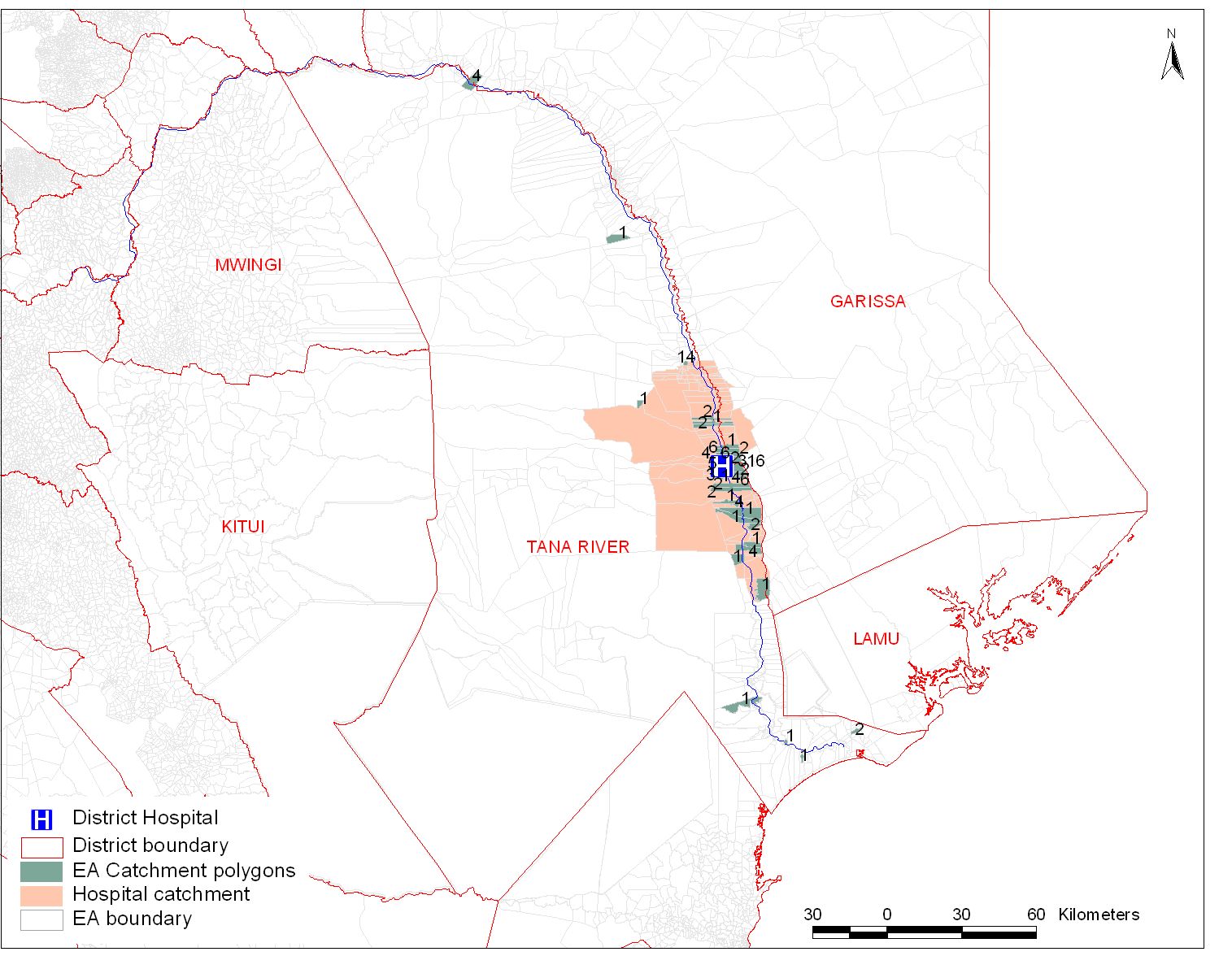

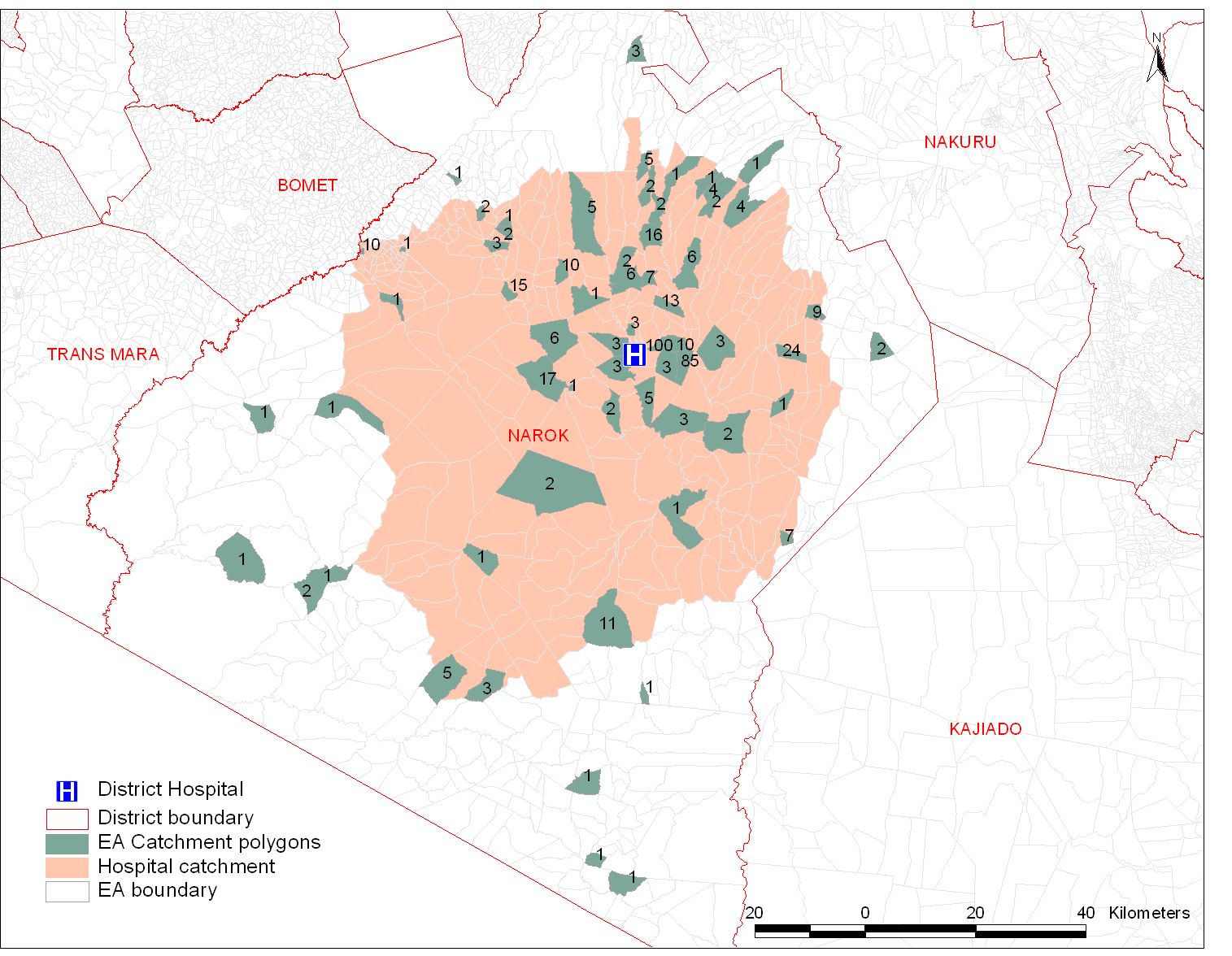
**

**a) Narok b) Hola**


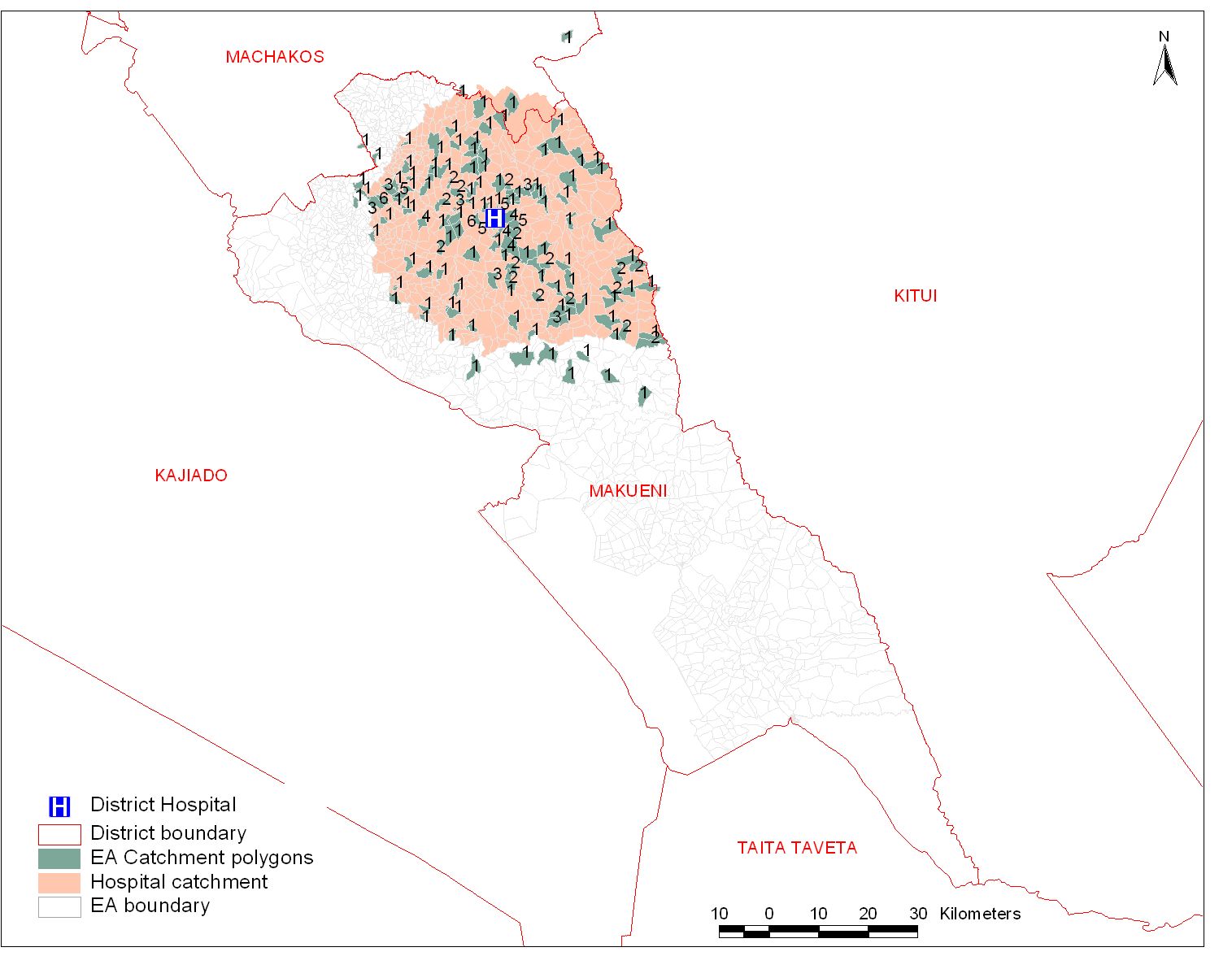

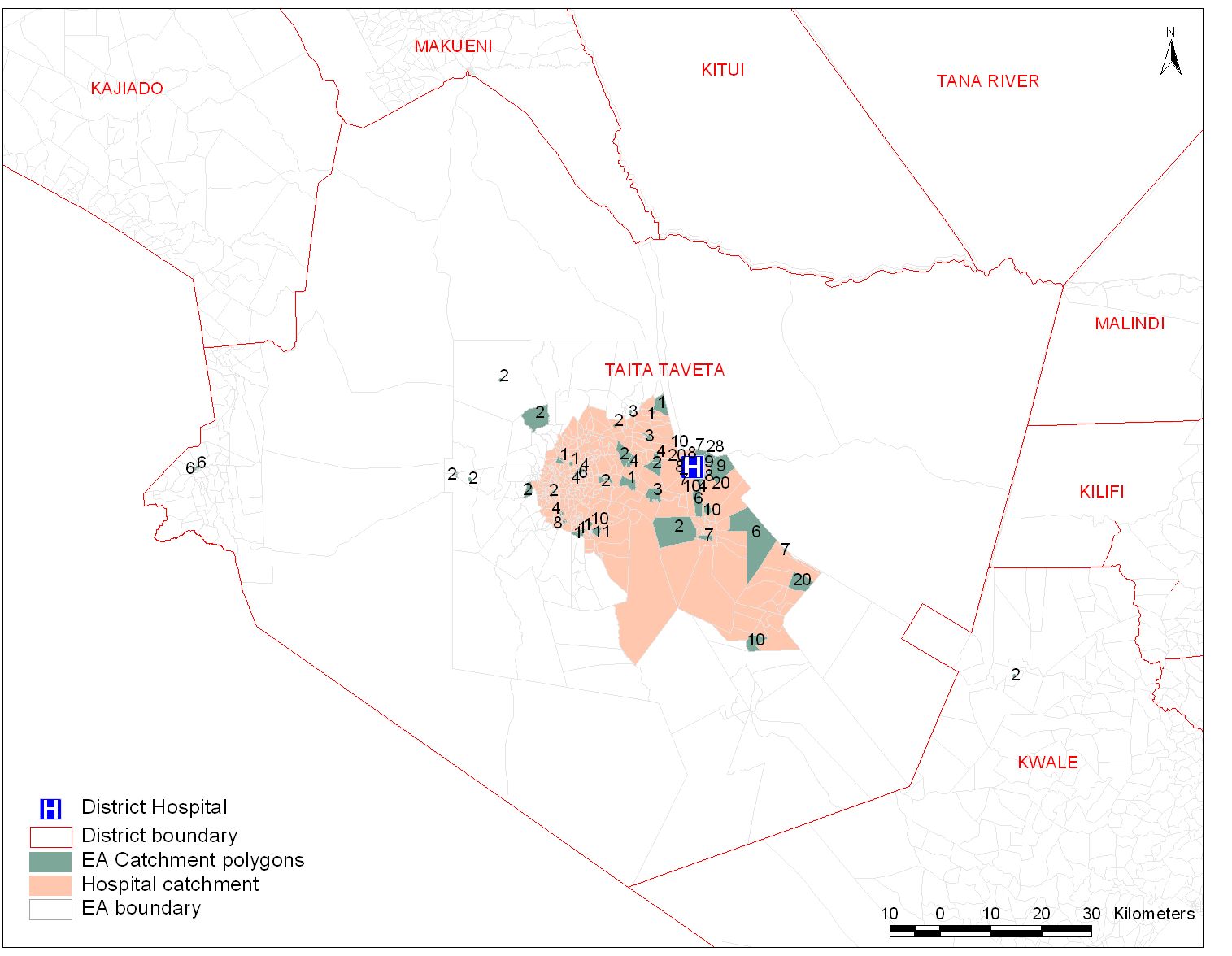
**IV. Arid and Semi-Arid areas:**

**c) Voi d) Makueni**


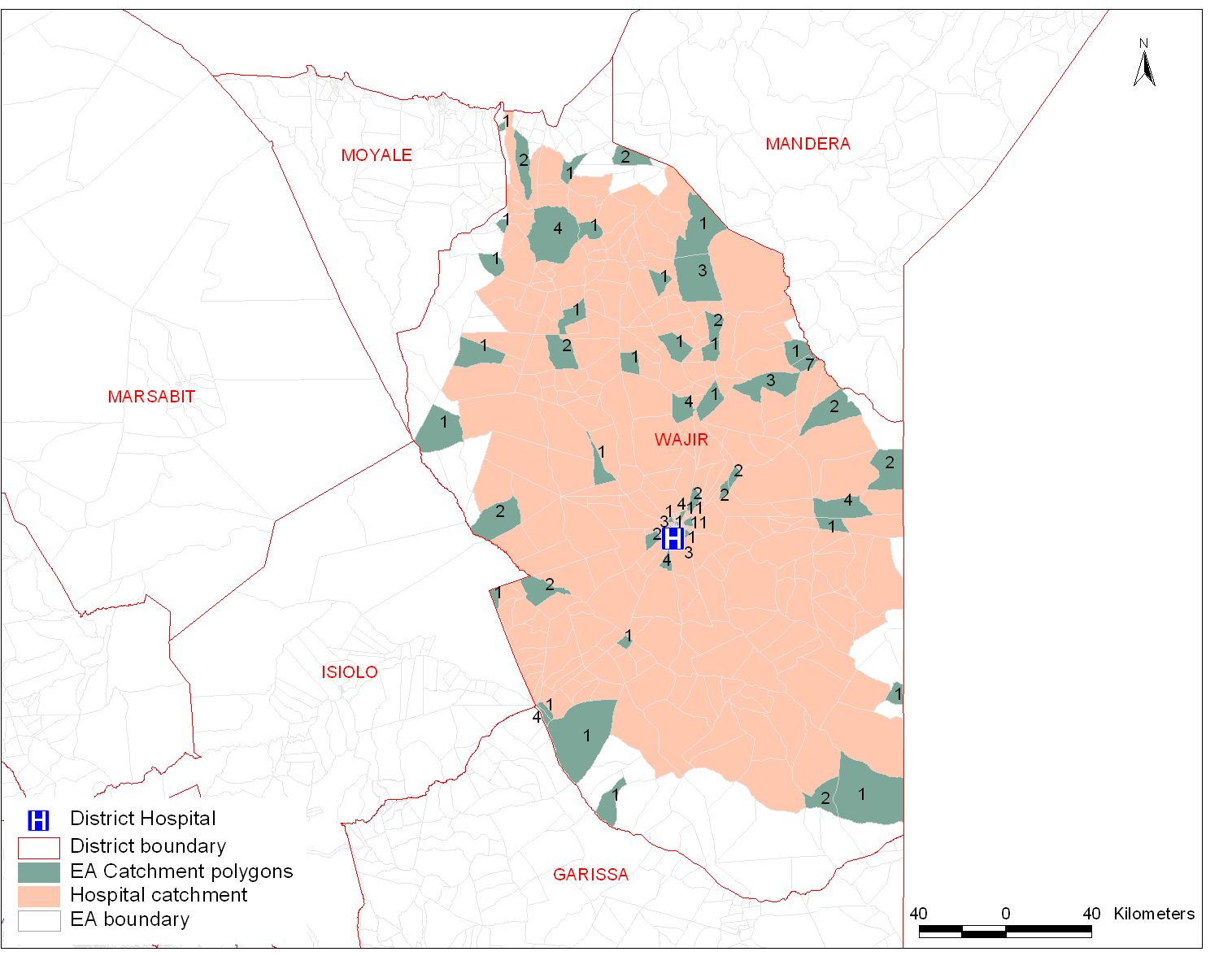
**IV. Arid and Semi-Arid areas:**

**e) Wajir**

**Rerences**

1. Noor AM, Gething PW, Alegana VA, Patil AP, Hay SI, Muchiri E, Juma E, Snow RW: **The risks of *Plasmodium falciparum* infection in Kenya in 2009**. *BMC Med* Submitted.

2. Central Bureau of Statistics: **1999 population and housing census: counting our people for development. Volume 2: Socio-economic profile of the people***.* Central Bureau of Statistics, Vol. 2: Ministry of Finance & Planning, GoK; 2001.

3. Tatem AJ, Noor AM, von Hagen C, Di Gregorio A, Hay SI: **High resolution population maps for low income nations: combining land cover and census in East Africa**. *PLoS ONE* 2007, **2**(12):e1298.
