# Supplementary material for: Malaria paediatric hospitalization between 1999 and 2008 across Kenya
Source: BMC Med. 2009 Dec 9;7:75. doi: 10.1186/1741-7015-7-75 (PMC2802588; doi:10.1186/1741-7015-7-75)
Supplement: Additional file 2 — Supplementary tables. [file 1741-7015-7-75-S2.DOCX]

**Supplementary Information 2**

**SI 2 Table 1:** Overall paediatric admission data for malaria and non-malaria at each of the 17 hospitals between1999-2008 expressed per 1000 children aged 0-14 years at risk per annum and 95% confidence intervals computed using a Poisson distribution.

| **Hospital location** | **Average annual malaria admission rate 1999 -2008**  **[95% CI]**  **(total number of malaria admissions)** | **Average annual non-malaria admission rate 1999 -2008**  **[95% CI]**  **(total number of non-malaria admissions)** |
| --- | --- | --- |
|  |  |  |
| **Western/Lakeside** |  |  |
| Busia | 9.91  [9.06-9.32]  (18,610) | 5.81  [5.71-5.92]  (11,772) |
| Bungoma | 5.65  [5.57-5.74]  (18,960) | 4.36  [4.29-4.44]  (14,634) |
| Bondo | 10.03  [9.81-10.26]  (7,552) | 7.28  [7.09-7.47]  (5,480) |
| Homa Bay | 12.09  [11.91-12.29]  (15,624) | 7.24  [7.10-7.39]  (9,356) |
| Kisumu | 11.63  [11.49-11.76]  (28,956) | 3.31  [3.24-3.38]  (8,237) |
| Siaya | 14.96  [14.73-15.19]  (16,689) | 8.26  [8.09-8.43]  (9,212) |
| **Highlands** |  |  |
| Kericho | 8.98  [8.85-9.12]  (17,697) | 6.67  [6.56-6.78]  (13,137) |
| Kisii | 21.01  [20.81-21.21]  (41,162) | 19.24  [19.05-19.44]  (37,699) |
| Kitale | 7.31  [7.23-7.39]  (31,432) | 3.83  [3.77-3.89]  (16,472) |
| **Coastal** |  |  |
| Kilifi | 7.85  [7.73-7.98]  (14,259) | 18.63  [18.43-18.82]  (33,813) |
| Malindi | 8.06  [7.89-8.24]  (8,109) | 16.26  [16.01-16.51]  (16,352) |
| Msambweni | 5.53  [5.40-5.67]  (6,292) | 8.88  [8.71-9.06]  (10,102) |
| **Arid & semi-arid** |  |  |
| Narok | 5.35  [5.21-5.50]  (5,126) | 7.03  [6.86-7.19]  (6,725) |
| Hola | 21.66  [21.13-22.20]  (6,261) | 8.25  [7.93-8.59]  (2,386) |
| Voi | 9.94  [9.71-10.18]  (6,869) | 7.49  [7.29-7.70]  (5,175) |
| Makueni | 1.89  [1.82-1.96]  (2,975) | 2.94  [2.86-3.03]  (4,639) |
| Wajir | 1.49  [1.44-1.55]  (3,321) | 2.66  [2.60-2.73]  (5,922) |

**SI 2 Table 2**: Temporally aggregated paediatric admission data for malaria each of the 17 hospitals between 1999-2002, 2003-2005 and 2006-2008 expressed per 1000 children aged 0-14 years at risk per annum and 95% confidence intervals computed using a Poisson distribution.

| **Hospital location** | **Average annual malaria admission rate 1999 -2002**  **[95% CI]**  **(total number of malaria admissions)** | **Average annual malaria admission rate 2003 -2005**  **[95% CI]**  **(total number of malaria admissions)** | **Average annual malaria admission rate 2006 -2008**  **[95% CI]**  **(total number of malaria admissions)** |
| --- | --- | --- | --- |
|  |  |  |  |
| **Western/Lakeside** |  |  |  |
| Busia | 6.30  [5.95-6.67]  (4,685) | 10.89  [10.44-11.35]  (6,700) | 10.76  [10.34-11.20]  (7,225) |
| Bungoma | 6.29  [6.01- 6.58]  (7,420) | 5.17  [4.93-5.42]  (5,252) | 5.39  [5.17-5.63]  (6,288) |
| Bondo | 6.90  [6.31- 7.53]  (1,979) | 13.06  [12.26-13.90]  (2,968) | 10.86  [10.15-11.60]  (2,605) |
| Homa Bay | 12.13  [11.52- 12.78]  (5,754) | 15.14  [14.48-15.82]  (5,933) | 9.26  (8.76-9.77]  (3,937) |
| Kisumu | 10.87  [10.46-11.29]  (10,156) | 17.40  (16.89-17.92]  (13,125) | 7.04  (6.73-7.36]  (5,675) |
| Siaya | 11.51  [10.88-12.16]  (5,006) | 16.54  [15.80-17.31]  (5553) | 17.70  [16.95-18.48]  (6,130) |
| **Highlands** |  |  |  |
| Kericho | 9.26  [8.83- 9.75]  (6,787) | 11.34  [10.88-11.81]  (6,749) | 6.51  [6.17-6.85]  (4,161) |
| Kisii | 24.26  [23.57-24.97]  (17,730) | 30.34  [29.60-31.09]  (17,845) | 8.91  [8.51-9.32]  (5,587) |
| Kitale | 7.63  [7.35-7.91]  (11,668) | 7.91  [7.65-8.18]  (10,267) | 6.51  [6.29-6.75]  (9,497) |
| **Coastal** |  |  |  |
| Kilifi | 12.78  [12.24-13.33]  (8,387) | 7.94  [7.55-8.35]  (4,337) | 2.56  [2.34-2.76]  (1,535) |
| Malindi | 10.45  [9.80-11.14]  (3,759) | 9.60  [9.01-10.21]  (2,907) | 4.31  [3.94-4.71]  (1,443) |
| Msambweni | 7.34  [6.84-7.88]  (3,064) | 5.06  [4.66-5.49]  (1,737) | 3.73  [3.67-4.38]  (1,491) |
| **Arid & semi-arid** |  |  |  |
| Narok | 3.48  [3.10-3.89]  (1,208) | 6.12  [5.64-6.63]  (1,779) | 6.69  [6.21-7.20]  (2,139) |
| Hola | 25.56  [23.66-27.57]  (2,646) | 27.78  [25.91-29.78]  (2,433) | 12.29  [11.15-13.53]  (1,182) |
| Voi | 10.96  [10.18-11.79]  (2,887) | 12.52  [11.71-13.38]  (2,608) | 6.26  [5. 70-6.86]  (1,374) |
| Makueni | 1.63  [1.43-1.85]  (943) | 2.62  [2.37-2.88]  (1,248) | 1.51  [1.33-1.70]  (784) |
| Wajir | 1.51  [1.33-1.71]  (989) | 1.46  [1.30-1.62]  (980) | 1.51  [1.37-1.66]  (1,352) |

**SI 2 Table 3**: Temporally aggregated paediatric admission data for non-malaria each of the 17 hospitals between 1999-2002, 2003-2005 and 2006-2008 expressed per 1000 children aged 0-14 years at risk per annum and 95% confidence intervals computed using a Poisson distribution.

| **Hospital location** | **Average annual non-malaria admission rate 1999 -2002**  **[95% CI]**  **(total number of non-malaria admissions)** | **Average annual non-malaria admission rate 2003 -2005**  **[95% CI]**  **(total number of non-malaria admissions)** | **Average annual non-malaria admission rate 2006 -2008**  **[95% CI]**  **(total number of non-malaria admissions)** |
| --- | --- | --- | --- |
|  |  |  |  |
| **Western/Lakeside** |  |  |  |
| Busia | 6.28  [5.93-6.65]  (4,654) | 5.89  [5.56-6.23]  (3,615) | 5.21  [4.92-5.52]  (3,502) |
| Bungoma | 5.60  [5.33-5.88]  (6,576) | 4.03  [3.82-4.24]  (4,102) | 3.42  [3.24-3.61]  (2,759) |
| Bondo | 9.11  [8.43-9.84]  (2,596) | 5.26  [4.76-5.80]  (1,199) | 7.05  [6.48-7.65]  (1,685) |
| Homa Bay | 8.23  [7.72-8.76]  (3,893) | 8.39  [7.90-8.90]  (3,278) | 5.12  [4.75-5.50]  (2,185) |
| Kisumu | 3.42  [3.18- 3.66]  (3,156) | 3.27  [3.05, 3.50]  (2,467) | 3.23  [3.02-3.44]  (2614) |
| Siaya | 8.99  [8.44-9.57]  (3,899) | 8.05  [7.54-8.60]  (2,706) | 7.55  [7.06-8.07]  (2,607) |
| **Highlands** |  |  |  |
| Kericho | 6.21  [5.86-6.58]  (4,536) | 7.42  [7.05-7.81]  (4,431) | 6.49  [6.16-6.84]  (4,170) |
| Kisii | 22.17  [21.49-22.86]  (16,297) | 16.07  [15.52-16.64]  (9,498) | 18.90  [18.31-19.49]  (11,904) |
| Kitale | 4.13  [3.93-4.43]  (6,321) | 3.21  [3.04-3.38]  (4,186) | 4.08  [3.90-4.26]  (5,965) |
| **Coastal** |  |  |  |
| Kilifi | 17.84  [17.20-18.49]  (11,760) | 18.74  [18.11-19.37]  (10,330) | 19.40  [18.80-20.02]  (11,723) |
| Malindi | 14.60  [13.82-15.41]  (5,280) | 17.52  [16.72-18.35]  (5,359) | 16.95  [16.20-17.72]  (5,713) |
| Msambweni | 9.96  [9.37-10.59]  (4,180) | 6.96  [6.49-7.46]  (2,395) | 9.49  [8.95-10.04]  (3,527) |
| **Arid & semi-arid** |  |  |  |
| Narok | 5.44  [4.96-5.95]  (1,882) | 7.01  [6.49-7.56]  (2,040) | 8.73  [8.18-9.31]  (2,803) |
| Hola | 7.97  [6.92-9.13]  (823) | 9.97  [8.86-11.18]  (874) | 7.07  [6.18-8.04]  (689) |
| Voi | 7.01  [6.38-7.68]  (1,841) | 7.51  [6.88-8.18]  (1,568) | 8.01  [7.38-8.69]  (1,766) |
| Makueni | 2.43  [2.18-2.69]  (1,409) | 2.85  [2.59-3.12]  (1,365) | 3.59  [3.31-3.88]  (1,865) |
| Wajir | 4.28  [3.96-4.61]  (2,708) | 2.48  [2.28-2.69]  (1,642) | 1.74  [1.59-1.89]  (1,573) |

**SI 2 Table 4**: Temporally aggregated paediatric admission data for all cause admissions each of the 17 hospitals between 1999-2002, 2003-2005 and 2006-2008 expressed per 1000 children aged 0-14 years at risk per annum and 95% confidence intervals computed using a Poisson distribution.

| **Hospital location** | **Average annual admission rate 1999 -2002**  **[95% CI]**  **(total number of admissions)** | **Average annual admission rate 2003 -2005**  **[95% CI]**  **(total number of admissions)** | **Average annual admission rate 2006 -2008**  **[95% CI]**  **(total number of admissions)** |
| --- | --- | --- | --- |
|  |  |  |  |
| **Western/Lakeside** |  |  |  |
| Busia | 12.58  [12.08-13.10]  (9339) | 16.78  [16.22-17.35]  (10,316) | 15.98  [15.46-16.51]  (10,727) |
| Bungoma | 11.89  [11.50-12.29]  (13,996) | 9.19  [8.88-9.52]  (9,355) | 8.81  [8.52-9.11]  (10,243) |
| Bondo | 16.01  [15.10-16.96]  (4,575) | 18.32  [17.37-19.31]  (4,167) | 17.90  [16.99-18.85]  (4,290) |
| Homa Bay | 20.36  [19.56-21.19]  (9647) | 23.53  [22.70-24.38]  (9,211) | 14.37  [13.76-15.01]  (6,122) |
| Kisumu | 14.29  [13.81- 14.77]  (13.312) | 20.67  [20.12-21.24]  (15,592) | 10.27  [9.90-10.66]  (8,289) |
| Siaya | 20.50  [19.65-21.36]  (8.905) | 24.59  [23.69-25.53]  (8,259) | 25.25  [24.35-26.18]  (8,737) |
| **Highlands** |  |  |  |
| Kericho | 15.47  [14.91-16.05]  (11,323) | 18.76  [18.16-19.37]  (11,180) | 13.00  [12.52-13.39]  (8,331) |
| Kisii | 46.43  [45.46-47.42]  (34,027) | 46.41  [45.48-47.35]  (27,343) | 27.80  [27.09-28.52]  (17,491) |
| Kitale | 11.75  [11.41-12.10]  (17,989) | 11.12  [10.81-11.43]  (14,453) | 10.59  [10.30-10.88]  (15,462) |
| **Coastal** |  |  |  |
| Kilifi | 30.61  [29.77-31.47]  (20,147) | 26.68  [25.94-27.43]  (14,667) | 21.96  [21.31-22.61]  (13,258) |
| Malindi | 25.05  [24.03-26.10]  (9,039) | 27.12  [21.12-28.15]  (8,266) | 21.26  [20.42-22.13]  (7,156) |
| Msambweni | 17.31  [16.96-19.02]  (7,244) | 12.02  [11.40-12.67]  (4,132) | 13.50  [12.86-14.16]  (5,018) |
| **Arid & semi-arid** |  |  |  |
| Narok | 8.91  [8.30-9.57]  (3,090) | 13.13  [12.42-13.87]  (3,819) | 15.42  [14.68-16.18]  (4,942) |
| Hola | 33.53  [31.35-35.82]  (3,469) | 37.75  [35.56-40.04]  (3,307) | 19.36  [17.89-20.92]  (1,871) |
| Voi | 17.97  [16.96-19.02]  (4,728) | 20.03  [19.00-21.11]  (4,176) | 14.27  [13.42-15.16]  (3,140) |
| Makueni | 4.06  [3.74-4.40]  (2,352) | 5.46  [5.11-5.84]  (2,613) | 5.10  [4.77-5.44]  (2,649) |
| Wajir | 5.79  [5.42-6.18]  (3,996) | 3.93  [3.68-4.20]  (2,622) | 3.25  [3.05-3.46]  (2,924) |
