# Supplementary material for: Malaria paediatric hospitalization between 1999 and 2008 across Kenya
Source: BMC Med. 2009 Dec 9;7:75. doi: 10.1186/1741-7015-7-75 (PMC2802588; doi:10.1186/1741-7015-7-75)
Supplement: Additional file 3 — ARMAX regression model coefficients of the monthly malaria incidence (1999-2008) on rainfall and non-malaria case incidence for each hospital site. [file 1741-7015-7-75-S3.DOC]

**Table: ARMAX regression model coefficients of the monthly malaria incidence (1999-2008) on rainfall and non-malaria case incidence for each hospital site.**

|  | **Bondo** | **Siaya** | **Kisumu** | **Homa Bay** | **Bungoma** | **Busia** | **Kilifi** | **Msambweni** | **Malindi** | **Kitale** | **Kisii** | **Kericho** | **Voi** | **Narok** | **Hola** | **Makueni** | **Wajir** |
| --- | --- | --- | --- | --- | --- | --- | --- | --- | --- | --- | --- | --- | --- | --- | --- | --- | --- |
|  |  |  |  |  |  |  |  |  |  |  |  |  |  |  |  |  |  |
| **marate_1000** |  |  |  |  |  |  |  |  |  |  |  |  |  |  |  |  |  |
| **L.rainfall** |  |  |  |  |  |  |  |  |  |  |  |  |  |  |  |  | 0.000391*** |
|  |  |  |  |  |  |  |  |  |  |  |  |  |  |  |  |  | (3.84) |
| **D.rainfall** |  |  |  |  |  | 0.000426* |  |  |  |  |  |  |  |  |  |  |  |
|  |  |  |  |  |  | (2.07) |  |  |  |  |  |  |  |  |  |  |  |
| **LD.rainfall** |  |  |  |  |  |  |  |  |  |  |  |  | 0.000940* |  |  |  |  |
|  |  |  |  |  |  |  |  |  |  |  |  |  | (2.08) |  |  |  |  |
| **L2D.rainfall** | 0.00128** | 0.00128*** | 0.00105*** | 0.000627 | 0.000372* | 0.000494 | 0.000892*** |  | 0.000816** | 0.000182 | 0.00203 | 0.000702 | 0.00251*** | 0.000282 | 0.00173 | 0.000265*** |  |
|  | (2.96) | (3.42) | (3.9) | (1.68) | (2.13) | (1.96) | (4.18) |  | (3.18) | (0.74) | (0.86) | (1.51) | (5.87) | (1.58 | (0.74) | (3.78) |  |
| **L3D.rainfall** |  |  |  |  |  |  |  | 0.000278 |  |  |  |  |  |  |  | 0.000302*** |  |
|  |  |  |  |  |  |  |  | (1.81) |  |  |  |  |  |  |  | (4.44) |  |
| **L4D.rainfall** |  |  |  |  |  |  |  |  |  |  |  |  |  |  |  | 0.000446*** |  |
|  |  |  |  |  |  |  |  |  |  |  |  |  |  |  |  | (8.13) |  |
| **D.nonmarate_1000** | 0.170 | 0.610*** | 0.769** | 0.495*** | 0.564*** | 0.681*** | 0.285* | 0.382*** | 0.272*** | 0.539* | 0.962*** | 1.346*** | 0.295 | 0.305*** | 0.347 | 0.501*** |  |
|  | (0.92) | (3.55) | (2.59) | (3.86) | (4.38) | (4.57) | (2.50) | (5.67) | (3.74) | (2.35) | (4.38) | (5.29) | (1.55) | (3.54) | (0.84) | (6.44) |  |
| **Nonmarate_1000** |  |  |  |  |  |  |  |  |  |  |  |  |  |  |  |  | -0.0384 |
|  |  |  |  |  |  |  |  |  |  |  |  |  |  |  |  |  | (-1.10) |
| **Constant** | 0.00503 | 0.0185 | 0.00233 | 0.000562 | 0.000135 | 0.00487** | -0.0117*** | -0.00435 | -0.00753* | -0.00229 | -0.0154 | -0.0046 | -0.00462 | 0.00135 | -0.0214 | -0.000112 | 0.124*** |
|  | (0.73 | (1.14) | (0.21) | (-0.09) | (0.06) | (2.96) | (-7.14) | (-1.25) | (-2.15) | (-0.19) | (-0.60) | (-0.51) | (-0.76) | (0.65) | (-0.54) | (-0.04) | (11.1) |
|  |  |  |  |  |  |  |  |  |  |  |  |  |  |  |  |  |  |
| **N** | 117 | 117 | 117 | 117 | 117 | 117 | 117 | 116 | 117 | 117 | 117 | 117 | 117 | 117 | 112 | 115 | 119 |
| **AIC** | 61.82 | 61.29 | 29.77 | 32.18 | -122.9 | -35.24 | 54.87 | -110.6 | 25.25 | -64.72 | 407 | 101 | 54.34 | -155.3 | 342.5 | -266.3 | -361.1 |
| **BIC** | 81.15 | 80.62 | 51.87 | 51.51 | -106.4 | -15.9 | 76.97 | -94.06 | 47.35 | -45.38 | 426.4 | 120.3 | 73.67 | -138.7 | 364.3 | -244.3 | -347.2 |

**Footnotes**

Marate_1000 - Malaria admission rates per 1000 population 0-14 years

Nonmarate_1000 - Non-Malaria admission rates per 1000 population 0-14 years

Regression coefficients with t statistics provided in the parentheses

Level of significance indicated by asterisks - * p<0.05, ** p<0.01, *** p<0.001

D – Differenced data (current value minus previous value to make data stationary)

L- Lag of 1 month

L2 - Lag of 2 months

L3 - Lag of 3 months

L4 - Lag of 4 months

Constant is the estimated mean outcome per month

AIC- Akaike Information Criterion

BIC - Bayesian information criterion
